# *In vivo* detection of HIV-1 antisense transcripts in untreated and ART-treated individuals

**DOI:** 10.1101/2024.12.06.627170

**Published:** 2025-02-25

**Authors:** Adam A. Capoferri, Rachel Sklutuis, Toluleke O. Famuyiwa, Sachi Pathak, Rui Li, Jason W. Rausch, Brian T. Luke, Rebecca Hoh, Steven G. Deeks, John W. Mellors, John M. Coffin, Jennifer L. Groebner, Fabio Romerio, Mary F. Kearney

**Affiliations:** 1 HIV Dynamics and Replication Program, National Cancer Institute, Frederick, MD, USA; 2 Department of Molecular and Comparative Pathobiology, Johns Hopkins University School of Medicine, Baltimore, MD, USA; 3 Leidos Biomedical Research, Inc, Frederick National Laboratories for Cancer Research, Frederick, MD, USA; 4 Department of Medicine, University of California, San Francisco, CA, USA; 5 Division of Infectious Diseases, Department of Medicine, University of Pittsburgh School of Medicine, Pittsburgh, PA, USA; 6 Department of Molecular Biology and Microbiology, Tufts University, Boston, MA, USA

## Abstract

Natural antisense transcripts are expressed in eukaryotes, prokaryotes, and viruses and can possess regulatory functions at the transcriptional and/or post-transcriptional levels. *In vitro* studies have shown that HIV-1 antisense transcripts (AST) promote viral latency through epigenetic silencing of the proviral 5′ long terminal repeat (LTR). However, expression of HIV-1 AST *in vivo* have not been convincingly demonstrated. Here, we used single RNA template amplification, detection, and sequencing to demonstrate expression of AST in unstimulated PBMC collected from people with HIV-1 (PWH). We found that AST had high genetic diversity that matched proviruses in cells from blood and lymph nodes. We measured a median of 26 copies of AST per 100 infected cells in PWH on ART and a median of 2 copies per 100 infected cells in PWH not on ART. The expression of HIV-1 AST *in vivo* is consistent with a potential regulatory role in regulation of HIV-1 expression.

Antisense transcripts (AST) are RNA molecules transcribed from the opposite strand of a protein-coding gene that can have protein-coding and/or non-coding activities (1). AST have been identified in eukaryotes, prokaryotes, and viruses and have been shown to possess regulatory functions at both the transcriptional and post-transcriptional levels via multiple mechanisms (2). AST have previously been documented to be encoded by several viruses that infect eukaryotes including members of the *Herpesviridae* (*e.g.* herpes simplex virus-1 and cytomegalovirus) (3, 4) and *Retroviridae* (5–7) families. One of the best characterized AST in a viral system is the *Hbz* gene in Human T-cell Leukemia Virus Type 1 (HTLV-1). The interplay between *Hbz* (antisense) and *Tax* (sense) RNA expression and their protein products is thought to modulate the regulation of cellular pathways that promote survival and proliferation of HTLV-1 infected cells, thereby influencing the progression into adult T cell leukemia/lymphoma or HTLV-1-associated myelopathy/tropical spastic paraparesis (8).

R.H. Miller first provided evidence of an antisense gene (named *asp*) overlapping the *env* gene in the HIV-1 genome (9). Several *in vitro* studies have since demonstrated the presence of a Tat-independent negative sense promoter in the HIV-1 3′ long terminal repeat (LTR) (5, 10, 11), which drives the expression of multiple antisense transcripts (12) with both protein coding (13) and non-coding functions (14). The presence of HIV-1 antisense protein (ASP)-specific antibodies in serum (15) and cytotoxic CD8+ T-lymphocytes (CTLs) in blood samples (16) have been detected from people living with HIV (PWH), thus providing indirect evidence for the expression of ASP *in vivo*. Detection of ASP has been reported in eight chronically infected lymphoid and myeloid cell lines during latent and productive infection (17). HIV-1 AST have been shown to inhibit viral replication and to promote and maintain latency in stably expressing CD4+ T cell lines (18, 19). Although *in vitro* studies suggest that AST function in viral latency, only a few reports have examined its expression *in vivo*. Zapata *et al.* reported low levels of AST in 3 PWH on antiretroviral therapy (ART) (>2 years with undetectable levels of viremia) using real-time PCR in unstimulated CD4+ T cells (19) and Mancarella *et al.* (20) detected AST in CD4+ T cells collected from both untreated and ART-treated PWH after *ex vivo* stimulation with anti-CD3/CD28. However, no studies have provided sequence evidence for expression of AST in unstimulated cells collected from PWH.

Therefore, we set out to determine if we could measure and genetically characterize HIV-1 AST in unmodified peripheral blood mononuclear cells (PBMC) collected from donors on and off ART, and to compare thier expression levels to that of HIV-1 sense transcripts in the same donors. Investigating expression of HIV-1 AST *in vivo* may contribute to our understanding of HIV-1 persistence and reveal new targets for controlling HIV-1 expression without ART.

## Results

### Participants and samples

To determine the levels of AST during chronic infection, PBMC were collected from 3 PWH on ART (21) and 5 PWH who were not on ART (Table 1) and who were enrolled either at University of California of San Francisco under the SCOPE trial (clinical trial # NCT00187512) or at University of Pittsburgh under the Optimization of Immunologic and Virologic Assays for HIV Trial (IRB# STUDY20040215). All donors provided written informed consent for the study. For one donor (PID 2669), samples collected at four longitudinal timepoints before and after an ART interruption and re-initiation were available.

Participants were mostly assigned male at birth (n=7/8). Race/ethnicity as reported by donors was Black (n=4/8), White (n=3/8), and Latino (n=1/8). Donors were diagnosed with HIV-1 subtype B with a minimum duration of infection median of 8 years [IQR 5–13 years] prior to sample collection, however this information was unknown for one participant (PID 2669), who was on study more than 5 years. Donors not on ART (untreated) were either ART naïve or not currently on an ART regimen due to a planned or unplanned interruption, and their plasma viremia levels were detectable (median 139,945 range: 128 – 275,685 HIV-1 RNA copies/mL). All but one of the samples collected from PWH on ART had undetectable levels of viremia (<50 HIV-1 RNA copies/mL) with durations of treatment ranging from 2 weeks to 12.8 years. The one sample with detectable viremia on ART (112 HIV-1 RNA copies/mL) was collected from PID 2669 2 weeks after ART re-initiation after an unplanned 4-week treatment interruption. Levels of plasma viremia were measured as HIV-1 RNA copies/mL by either Abbott^®^ real-time assay or COBAS^®^ HIV-1 test.

### HIV-1 AST were detected in ART-treated and untreated PWH, with higher levels in samples collected during ART

Our methods for detecting and measuring levels of HIV-1 AST were modified from Wiegand *et al.* (22), Capoferri *et al. (*23*)*, and Zapata *et al.* (19) and are described in detail in the Supplementary Materials. Briefly, to measure levels of HIV-1 AST *in vivo*, we extracted total cell-associated RNA from unmodified donor PBMC with known estimated numbers of HIV-1 infected cells (24), synthesized cDNA using participant-specific exogenous oligo-tagged gene-specific primers targeting AST in the *env* coding region (19), and quantified the number of AST in each sample with participant-specific anti-*env* primers and probes (primer/probe sequences in Tables S1–S5) in a digital PCR format. Prior to testing the donor PBMC, we optimized the AST assay on antisense RNA in the ACH-2 cell line and found these cells to express a median of 41 AST per 100 infected ACH-2 cells (Supplementary Materials). Extensive controls were performed to ensure complete degradation of HIV-1 DNA (22, 23) and to determine the cut-off for HIV-1 AST (details in Supplementary Materials). Equal numbers of no reverse transcriptase wells (negative controls) and experimental wells were included on each PCR plate to ensure no HIV-1 DNA contamination.

**Fig. 1.**
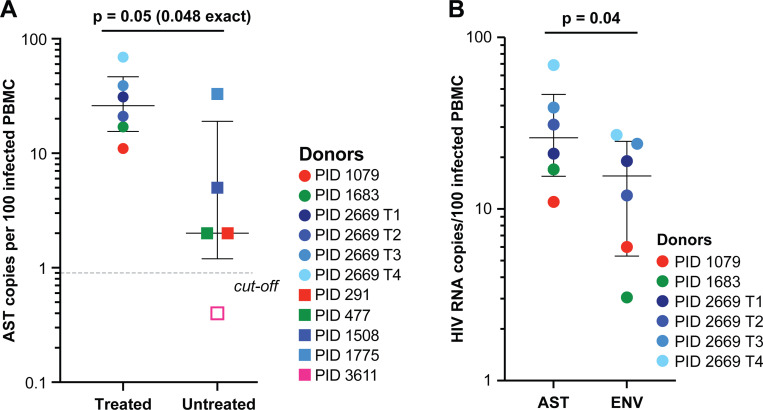
Levels of HIV-1 AST and sense *env* transcripts in PWH. (**A**) Levels of HIV-1 AST in ART-treated and untreated PWH. PWH on ART (circles) and PWH not on ART (squares), each color represents an individual, and PID 2669 has multiple shades of one color to indicate longitudinal sampling. P value determined with Mann-Whitney U-test. Open shape indicates a sample where AST was detected but was below the assay cut-off. The cut-off was determined as the number of positive RT-wells per number of infected cells assayed across 16 replicates of 100 ACH-2 in a background of 10^5^ CEM cells (see Supplementary Materials). (**B**) Detection of AST vs. sense *env* in ART-treated PWH. P value determined with paired t-test.

AST were detected in all 8 donors (Fig. 1A & Table S6). The level of AST in ART-treated samples was a median of 26 copies/100 infected PBMC [IQR 16–47] and the level in untreated samples was a median of ≤ 2 copies/100 infected PBMC [IQR 1–19] (p=0.05, exact p=0.048, Mann-Whitney U-test). Since PID 2669 was overrepresented in the treated group, we also performed the comparison by aggregating the AST in the 2 timepoints prior to the ART interruption, to include only one data point per donor. The re-analysis resulted in a median of 17 AST/100 infected PBMC in the donors on ART vs. ≤ 2 AST/100 infected PBMC in the untreated donors (p=0.21, Mann-Whitney U-test). In PID 3611 (untreated), only 1 AST molecule was detected in 244 infected cells, which was below the cut-off for the assay (details for cut-off in Supplementary Materials), indicating that the AST in this donor sample was ≤ 0.4 copies/100 infected PBMC. In PID 1775 (untreated), detection of 33 copies/100 infected PBMC was an outlier among the other untreated PWH (≤ 0.4–5 copies/100 infected PBMC), as confirmed by the Grubbs’ Test for Outliers (G=1.8, α=0.05) (25). In PID 2669 (treated), AST were detected in samples collected at all 4 timepoints: 4.3 and 5.5 years after ART initiation and 2 weeks and 4 weeks after ART re-initiation following a brief treatment interruption. Samples after ART re-initiation had higher levels of AST than samples collected prior to ART interruption (49% of 133 infected PBMC vs. 25% of 155 infected PBMC) (p=3.4×10^−9^, binomial test).

### HIV-1 AST were detected at modestly higher levels than HIV-1 sense env transcripts in samples on ART

In the samples on ART, we also measured the levels of sense *env* RNA targeting the same sub-genomic region targeted for AST (Fig. 1B). Levels of sense *env* RNA (aggregate of unspliced and partially spliced) were quantified similarly as above but as a separate reaction from HIV-1 AST. Levels of AST were modestly higher than sense *env* RNA (median 26 copies/100 infected PBMC [IQR 16–47] vs. median 16 copies/100 infected PBMC [IQR 5–25]) (paired t-test t(5)=2.79, p=0.04).

### Long fragments of HIV-1 AST were amplified from single infected cells from donors on ART

Because of the higher levels of AST in the donors on ART, we were able to amplify longer fragments in this subset. We amplified 1.7-kb fragments of AST spanning from the negative sense promoter in the 3′ LTR to *env* (referred to here as “long AST”). Using a modified version of the CARD-SGS assay (22, 23), we used the sequence data to estimate the fraction of infected cells with “long AST” and the levels of AST in single infected cells (Table S2; details in Supplementary Materials). We found that a median of 4.1% [IQR 1.6 – 5.2%] of infected PBMC had detectable levels of “long AST” with a median of 1.1 copies/cell [IQR 1.0–1.7] at a given point in time, indicating that the frequency of detection of “long AST” was lower than the short AST fragments detected by the digital PCR approach (above). In PID 2669, we found no significant difference in the fraction of cells with “long AST” or the levels of AST in single infected cells across the four time points (Kruskal-Wallis test, H(3)=3.02, p=0.39). Additionally, we found no significant difference when we aggregated the samples prior to ART interruption and after ART-reinitiation (p=0.83, Mann-Whitney U-test).

### Phylogenetic analysis of AST reveals that their expression originates from a diverse population of proviruses

**Fig. 2.**
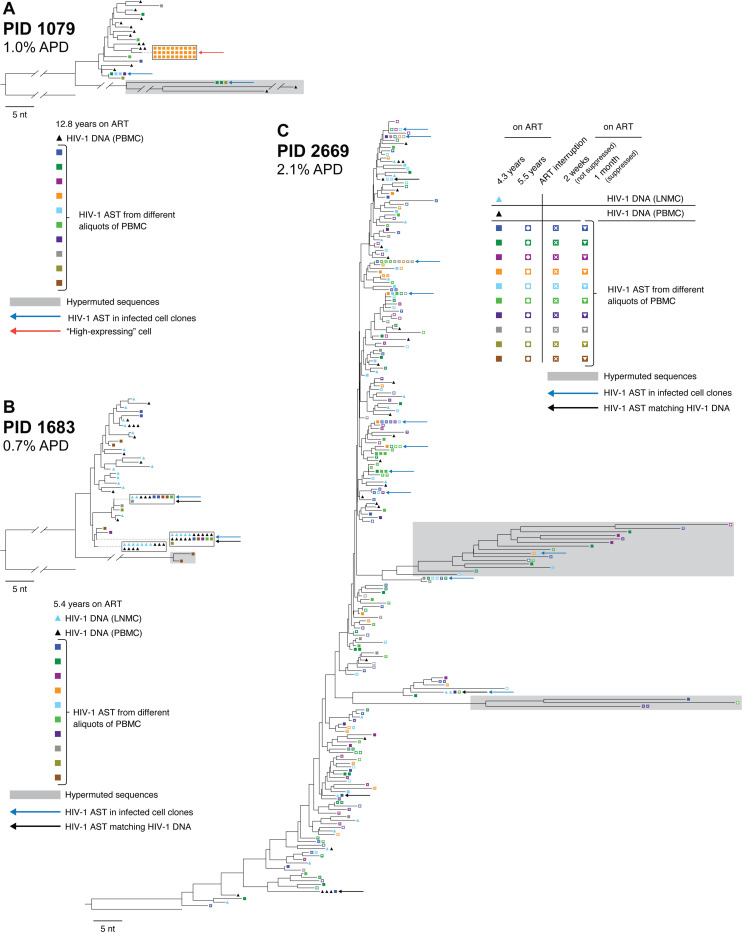
Distance trees of AST in donors on ART. HIV-1 AST sequenced using the modified CARD-SGS assay (Supplementary Materials). Percent average pairwise distance (APD) was calculated without hypermutants. HIV-1 AST sequences were aligned with the HIV-1 DNA *env* sequences (previously reported in McManus *et al*. (21)). Black triangles show proviruses from PBMC, light blue triangles show proviruses from lymph node mononuclear cells (LNMC), and squares are the intracellular AST from PBMC with each color representing a different aliquot of PBMC. Blue arrows indicate identical AST sequences found in >1 aliquot of PBMC, black arrows indicate identical AST sequences that matched either PBMC or LMNC HIV-1 DNA, and the red arrow indicates a “high AST-expressing” cell. Predicted hypermutant sequences are shaded in gray. Trees are rooted on consensus subtype B *env*.

Having successfully amplified the 1.7-kb segments of HIV-1 AST in the donors on ART by RT-PCR, we sequenced the resulting PCR products and performed phylogenetic analyses to compare the genetics of the AST to the proviruses in the same population of infected cells (Fig. 2). Standard HIV-1 *env* DNA single-genome sequencing using PBMC from all 3 donors and lymph node mononuclear cells (LNMC) from 2 of the donors was performed previously (21) and the data used here as a reference. Neighbor-joining trees were reconstructed using HIV-1 *env* DNA from PBMC and LNMC and ~1.0-kb of the AST in the same genetic region. Trees were rooted on the HIV-1 subtype B consensus sequence. Symbols on the trees show HIV-1 *env* DNA from PBMC (black triangles), HIV-1 *env* DNA from LNMC (blue triangles), and AST (multicolored squares where each color is obtained from a different aliquot of PBMC). AST matching HIV-1 *env* DNA in PBMC or LNMC are indicated with black arrows; AST from infected probable T cell clones are indicated with blue arrows. In PID 1079, a cell expressing high levels of AST is indicated with a red arrow. The genetic diversity of AST was measured by average pairwise distance (APD) with predicted hypermutant sequences removed (26).

We detected a high genetic diversity of AST (ranging from 0.7–2.5%) matching a diverse population of proviruses in both PBMC and in LNMC (1.1–2.1%), indicating that a wide variety of proviruses can express AST (Fig. 2). In one aliquot of PBMC from PID 1079, we found a rake of 30 identical AST, suggesting that they may have originated from the same infected cell (red arrow, Fig. 2A). Further, we found identical AST across multiple aliquots of infected PBMC in all 3 donors, indicating that these AST may have originated from infected T cell clones (blue arrows). In PID 1683, we also found identical PBMC AST matching proviruses in both PBMC and LNMC (Fig. 2B). While we did not directly assess the presence of AST in LNMC due to limited sampling with fine needle aspirates, these data suggest that AST may be expressed in cell clones that are present in tissues as well as in blood. AST were highly diverse in all 4 samples collected from PID 2669: T1 (2.9% APD), T2 (2.4%), T3 (2.6%), and T4 (2.4%). We also identified 11 likely T cell clones that contained some cells with AST (blue arrows). Eight of the 11 clones were found to persist across multiple timepoints, mostly either before or after the ART interruption, and only very rarely persisting both before and after the interruption, suggesting that ART interruption may influence the populations of T cells that express HIV-1 AST (Fig. 2C).

### AST can be detected in gag, pol, and env coding regions in vivo

*In vitro* studies have detected AST of varying lengths, including “full-length” (*i.e.*, from the U3/R of the 3′ LTR to *gag*) (5, 11, 12) (Fig. S1A, Supplementary Materials). We asked if AST could be detected, not only in *env*, but in other HIV genomic regions *in vivo*. We measured AST in *gag* (HXB2: 764–2,281) (27), *gag*/*pol* (HXB2: 1,849–3,500) (22) and *pol*/*vif* (HXB2: 3,996–5,270) in one donor sample (PID 2669 Timepoint #4) (28) (Fig. S3A). Cell-associated RNA from three aliquots of ~90 infected PBMC was extracted and cDNA targeting the antisense strand of *gag*, *gag/pol*, and *pol/vif* was synthesized using exogenous oligo-tagged gene-specific primers, followed by endpoint PCR amplification and Sanger sequencing (primers in Table S1,4). We detected AST in all genomic regions assayed. However, we found lower levels of genetic diversity for AST in *gag*, *gag/pol*, and *pol/vif* regions compared to AST in *env* at the same time points (1.5% vs 3.2%) (Fig. S3B). Comparable to levels of “long AST” in *env* (1.7kb described above), levels of AST in *gag*, *gag/pol*, and *pol/vif* were 1.0, 1.1, and 1.0 copies/cell, respectively. Although the sub-genomic regions cannot be genetically linked, these findings suggests that AST may span the entire HIV-1 genome in some infected cells.

## Discussion

In this study, we used established quantitative PCR methods (19), extensive positive and negative controls, and sequencing to demonstrate the expression of HIV-1 antisense RNA in people living with HIV who are either untreated or are on long or short-term ART. Prior to our study, HIV-1 antisense RNA had been shown to promote and maintain viral latency in stably expressing cell lines by recruiting the enhancer of zeste homolog 2 (EZH2), a core component of the polycomb repressive complex 2 (PRC2), to the HIV-1 5′ LTR (18, 19). Recruitment of EZH2 catalyzes trimethylation of lysine 27 on histone H3 (H3K27me3), a suppressive epigenetic mark that promotes nucleosome assembly and suppression of viral transcription. HIV-1 AST are inefficiently polyadenylated and predominately retained in the nucleus to act as a lncRNA (29). Although HIV-1 AST have been shown to inhibit viral replication and promote the establishment and maintenance of latency *in vitro* (19, 30), studies investigating AST *in vivo* have been limited. Therefore, we sought to determine if AST are expressed *in vivo* in untreated and/or ART-treated PWH, to quantify their levels in bulk and in single infected cells, and to characterize their genetics. To achieve this, we used a digital PCR assay for the detection and quantification of AST in unstimulated PBMC and we used a modified version of CARD-SGS (22, 23) to measure the fraction of infected cells with longer fragments of AST (1.7kb from 3′ LTR to *env*), the levels of AST in single infected cells, and the genetic diversity of AST in the unstimulated PBMC populations. Although methods for the CARD-SGS assay have been published previously (22, 23), we included detailed methods in the Supplementary Materials.

We detected HIV-1 AST in all 8 donors independent of treatment status. However, we found 13-fold higher levels of AST in PBMC samples collected on ART compared to those not on ART, perhaps consistent with AST functioning in the maintenance of viral latency (19, 30). An alternative interpretation could be that, in untreated donors, there are higher levels of transcription from the 5′ LTR. RNA polymerase collision (*i.e.*, transcriptional interference, either “sitting duck” or “roadblock”) or RNA:RNA hybrid formation between 5′ LTR-driven and 3′ LTR-driven transcription may suppress the expression of AST. Except for one donor on ART (PID 1079) where we identified one cell that may have had about 30 copies of AST, the level of AST in single cells was very low, with a mean of 1.2 copy/cell (range 1–30 copies/cell). The low levels of HIV-1 AST are consistent with those reported for other eukaryotic AST. In about 25% of protein-coding genes, AST are expressed at approximately 1 or a few copies per cell at any given time (31). In contrast, some AST can be expressed at very high levels, as observed for human *MALAT1* at about 150 TPM (32). This range of antisense lncRNA expression has been well documented across cellular and tissue types in the Functional Annotation of the Mammalian Genome (FANTOM) (33–35), the Genotype-Tissue Expression (GTEx) consortium (32), the Encyclopedia of DNA Elements (ENCODE) project (31), and the Long non-coding RNA Knowledgebase (36).

In the 3 donors on ART, we amplified and sequenced the 1.7-kb fragment of HIV-1 AST overlapping *env* from about 80 infected cells per sample. We found high genetic diversity of the AST including matches to proviruses in peripheral blood and lymph nodes. In some instances, the AST matched both PBMC and LNMC proviral DNA, suggesting that at least some cells in infected CD4+ T cell clones can express HIV-1 AST. In the one donor on ART with samples from multiple timepoints (PID 2669), we identified probable T cell clones with detectable levels of AST that persisted over time, and one that matched proviral DNA found in both PBMC and LNMC. Interestingly, although identical AST were found across the timepoints before the ART interruption and across the timepoints after the ART interruption and re-initiation, only rarely were identical AST detected both before and after the interruption, suggesting that the treatment interruption may have influenced the populations of T cell clones expressing HIV-1 AST. Further supporting the influence of ART interruption on AST expression was the observation that levels of AST were significantly elevated in samples collected within weeks after ART re-initiation relative to samples collected on longer-term ART (4–5 years on ART). It is possible that levels of AST in donors after ART re-initiation may be different than in long-term treated individuals or that relatively short-lived latently infected cells express AST at higher levels, thus, the effect of ART interruption on levels of AST should be further investigated. Together these data show that HIV-1 AST expression, although typically at low levels, can be detected in probable infected T cell clones in both blood and tissues.

Previous *in vitro* studies have reported multiple HIV-1 AST species using northern blot and 5′ Rapid Amplification of cDNA Ends (5′ RACE): Class I (10kb) (12), II (5.5kb) (11), III-iii (3kb) (12), and IV-ii (2kb) (5) (Fig. S1A). Our detection of HIV-1 AST in genes other than in *gag*, *gag/pol*, and *pol/vif* at levels comparable to those we observed in *env*, together with the work by Kobayashi-Ishihara *et al*. (12), suggest that we detected numerous Class I transcripts, which are full-length, expressed at low levels, and not typically polyadenylated. Relatedly, it is important to note that neither our digital PCR nor our single-genome sequencing assays targeting *env* is informative as to which Class the identified AST transcripts belong.

There are some limitations of this study to consider. Due to the fragility and sparsity of HIV-1 AST, we almost certainly underestimate their frequency. We cannot rule out that HIV-1 AST are short-lived and may not always be detectable. Similarly, there is a potential loss of AST from inefficient reverse transcription due to the thermal stability of secondary and tertiary RNA structures (although the high temperature denaturing step leading into cDNA synthesis is designed to help mitigate such effects), or during the sodium acetate/ethanol precipitation of the antisense cDNA. These recovery limitations are supported by the controls included in the Supplementary Materials demonstrating recoveries of only ~50% for AST spiked samples. Detecting a single copy of HIV-1 AST naturally presents challenges, particularly in determining if the molecules are due to expression by an HIV-1 or host promoter. Readthrough generation of HIV-1 AST can occur when the provirus integrates opposite to a host gene promoter, allowing transcription into the HIV-1 3′ LTR to produce AST. Previous research identifying the transcription start site of HIV-1 AST *in vitro* used infected cell lines and 5′ RACE (11, 12). We attempted 5′ RACE on our donor samples to determine if the HIV-1 AST originated from an HIV-1 or host promoter at a single RNA molecule level, but without success due to limitations in assay sensitivity. More sensitive 5′ RACE technologies are needed to determine the transcription start site of single RNA molecules *in vivo*, including HIV-1 AST. However, our finding that there are differences in levels of AST across different cells of a T cell clone may favor expression from the HIV 3′ LTR promoter.

This study is the first to show, with sequence confirmation, the expression of HIV-1 AST in PWH. Additional studies are needed to confirm our observation of higher levels of AST in PWH on ART vs. PWH who are not on ART, and to determine if HIV-1 AST *in vivo* are driven by viral or host promoters. This study, together with those showing the role of HIV-1 AST in inducing and maintaining HIV-1 latency *in vitro* and *ex vivo* (Li *et al.* Romerio, submitted), highlights a previously underexplored potential determinant of HIV-1 persistence both before and during ART, and may lead to new directions for the development of approaches to controlling HIV-1 viremia without ART.

## Supplementary Material

Supplement 1

## Figures and Tables

**Fig. 3.**
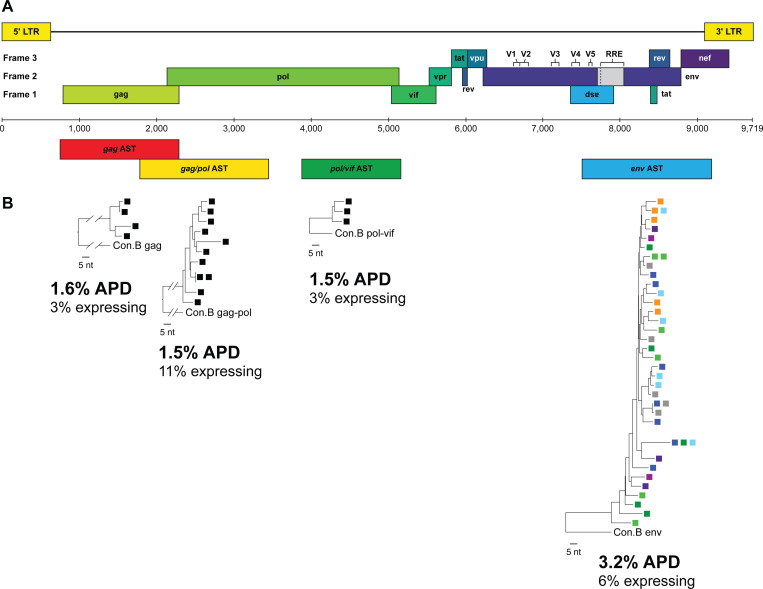
Detection of antisense transcripts along the proviral genome. (**A**) HIV-1 genome map indicating sub-genomic regions for AST amplification. (**B**) Modified AST single-genome sequencing using exogenous oligo-tagged primers to synthesize cDNA and to PCR amplify antisense transcripts along the genome. RNA from aliquots of 90 infected PBMC were extracted from PID 2669 Timepoint #4 for each sub-genomic region (anti-*gag*, *gag/pol*, *pol/vif*) and denoted by a single colored square. p-distance neighbor-joining phylogenetic reconstruction of positive amplicons were generated and percent APD and fraction of expressing cells are reported. The AST tree for anti-*env* is extracted from Fig. 2C, where up to 10 aliquots of PBMC were used. Each color square represents a different aliquot of PBMC from Timepoint #4.

**Table 1.** Donor demographics.

Group	Participant Identifier (PID)	Cohort Location	Age (years)	Sex^A^	Race/Ethnicity^B^	Minimum duration of infection (years)	HIV plasma RNA copies/mL at sampling^C^	ART regimen at sampling^D^	Duration on ART at sampling
Donors on ART	1079	SCOPE, USCF	64	M	Latino	17.0	<50	FTC/TDF, ETV	12.8 years
	1683	SCOPE, USCF	45	M	White	8.0	<50	FTC/TDF, DRV/r	5.4 years
	2669	SCOPE, USCF	50 51 56 56	M	White	Unknown	<50	ABC/3TC/DTG	4.3 years
							<50		5.5 years
							112		2 weeks^E^
							<50		1 month^E^
Untreated	291	University of Pittsburgh	53	M	Black	9.0	184,243	Not on ART	0^F^
	477	University of Pittsburgh	26	F	Black	5.0	139,845	Not on ART	0^F^
	1508	SCOPE, USCF	53	M	Black	30.0	128	Not on ART	0^F^
	1775	SCOPE, USCF	37	M	White	5.0	1,704	Not on ART	0^F^
	3611	SCOPE, USCF	37	M	Black	4.0	275,685	Not on ART	0^F^

# Initiated ART following 4 week ART interruption

A Sex assigned at birth: M (male) and F (female)

B Race/Ethnicity reported by donor

C Level of plasma viremia was determined by either Abbott^®^ Real-time HIV-1 Assay (SCOPE, UCSF) or COBAS^®^ HIV-1 Test (University of Pittsburgh)

D ABC (abacavir), DTG (dolutegravir), DRV (darunavir), ETV (etravirine), FTC (emtricitabine), TDF (tenofovir disoproxil fumarate),/r (ritonavir-boosted), 3TC (lamivudine)

E After 5.5 years on ART, the participant had an unexpected ART interruption for approximately 4 weeks. They reinitiated ART with the first timepoint post-ART interruption at 2 weeks with low but detectable HIV-1 plasma viremia. Then 1 month post-ART interruption with plasma viremia suppressed

F Donors were not on ART

**Table 2.** Fraction of infected cells with HIV-1 “long AST” in donors on ART.

Participant Identifier (PID)	Duration on ART at sampling	Estimated number of infected cells assayed	Number of HIV-1 AST sequences obtained	Estimated number of infected cells with HIV-1 AST^A^	Estimate % of infected cells with HIV-1 AST	Average number of HIV-1 AST copies per cell [range]^B^
1079	12.8 years	720	43	12	1.7	3.6 [1–30]
1683	5.4 years	1,880	22	20	1.1	1.1 [1–2]
2669	4.3 years	1,440	67	63	4.4	1.1 [1–3]
	5.5 years	1,040	54	52	5.0	1 [1–2]
	2 weeks^#^	2,000	86	74	3.7	1.2 [1–3]
	1 month	720	41	40	5.6	1 [1–2]
**Median** **IQR**		**1,240** **720–1,910**	**49** **36–72**	**46** **18–66**	**4.1** **1.6–5.2**	**1.1** **1.0–1.7**

A Assuming that identical sequence are from a single cell.

“long AST” = 1.7kb from 3ʹ LTR to *env*

A Assumes AST with identical sequences are produced in the same single infected cell

B Cells without HIV-1 AST were excluded

# Not fully suppressed after 2 weeks on ART following 4-week unplanned ART interruption (ATI). PID 2669 was on ART for 5.5 years prior to an ATI. Samples were collected at 4.3 years and 5.5 years prior to the ATI and at 2 weeks and 1 month after ART re-initiation after the ATI

## Data Availability

All sequence data are available on GenBank (in process of submission). Sequences from the donors on ART were previously published by McManus and colleagues and can be found in (21).

## References

[1] Khorkova O., Myers A. J., Hsiao J., Wahlestedt C., Natural antisense transcripts. Hum Mol Genet 23, R54–63 (2014). 24838284 10.1093/hmg/ddu207 PMC4170719

[2] Li R., Sklutuis R., Groebner J. L., Romerio F., HIV-1 Natural Antisense Transcription and Its Role in Viral Persistence. Viruses 13, (2021). 10.3390/v13050795 PMC8145503 33946840

[3] Kondo K., Xu J., Mocarski E. S., Human cytomegalovirus latent gene expression in granulocyte-macrophage progenitors in culture and in seropositive individuals. Proc Natl Acad Sci U S A 93, 11137–11142 (1996). 8855322 10.1073/pnas.93.20.11137 PMC38297

[4] Zwaagstra J., Ghiasi H., Nesburn A. B., Wechsler S. L., In vitro promoter activity associated with the latency-associated transcript gene of herpes simplex virus type 1. J Gen Virol 70 ( Pt 8), 2163–2169 (1989). 2549185 10.1099/0022-1317-70-8-2163

[5] Michael N. L., Vahey M. T., d’Arcy L., Ehrenberg P. K., Mosca J. D., Rappaport J., Redfield R. R., Negative-strand RNA transcripts are produced in human immunodeficiency virus type 1-infected cells and patients by a novel promoter downregulated by Tat. J Virol 68, 979–987 (1994). 8289399 10.1128/jvi.68.2.979-987.1994 PMC236536

[6] Rasmussen M. H., Ballarín-González B., Liu J., Lassen L. B., Füchtbauer A., Füchtbauer E. M., Nielsen A. L., Pedersen F. S., Antisense transcription in gammaretroviruses as a mechanism of insertional activation of host genes. J Virol 84, 3780–3788 (2010). 20130045 10.1128/JVI.02088-09 PMC2849499

[7] Larocca D., Chao L. A., Seto M. H., Brunck T. K., Human T-cell leukemia virus minus strand transcription in infected T-cells. Biochem Biophys Res Commun 163, 1006–1013 (1989). 2476979 10.1016/0006-291x(89)92322-x

[8] Bangham C. R. M., Miura M., Kulkarni A., Matsuoka M., Regulation of Latency in the Human T Cell Leukemia Virus, HTLV-1. Annu Rev Virol 6, 365–385 (2019). 31283437 10.1146/annurev-virology-092818-015501

[9] Miller R. H., Human immunodeficiency virus may encode a novel protein on the genomic DNA plus strand. Science 239, 1420–1422 (1988). 3347840 10.1126/science.3347840

[10] Bentley K., Deacon N., Sonza S., Zeichner S., Churchill M., Mutational analysis of the HIV-1 LTR as a promoter of negative sense transcription. Arch Virol 149, 2277–2294 (2004). 15338321 10.1007/s00705-004-0386-8

[11] Landry S., Halin M., Lefort S., Audet B., Vaquero C., Mesnard J. M., Barbeau B., Detection, characterization and regulation of antisense transcripts in HIV-1. Retrovirology 4, 71 (2007). 17910760 10.1186/1742-4690-4-71 PMC2099442

[12] Kobayashi-Ishihara M., Yamagishi M., Hara T., Matsuda Y., Takahashi R., Miyake A., Nakano K., Yamochi T., Ishida T., Watanabe T., HIV-1-encoded antisense RNA suppresses viral replication for a prolonged period. Retrovirology 9, 38 (2012). 22569184 10.1186/1742-4690-9-38 PMC3410806

[13] Gholizadeh Z., Iqbal M. S., Li R., Romerio F., The HIV-1 Antisense Gene ASP: The New Kid on the Block. Vaccines (Basel) 9, (2021). 10.3390/vaccines9050513 PMC8156140 34067514

[14] Ma G., Yasunaga J., Matsuoka M., Multifaceted functions and roles of HBZ in HTLV-1 pathogenesis. Retrovirology 13, 16 (2016). 26979059 10.1186/s12977-016-0249-x PMC4793531

[15] Caetano D. G., Napoleão-Pêgo P., Villela L. M., Côrtes F. H., Cardoso S. W., Hoagland B., Grinsztejn B., Veloso V. G., De-Simone S. G., Guimarães M. L., Patterns of Diversity and Humoral Immunogenicity for HIV-1 Antisense Protein (ASP). Vaccines (Basel) 12, (2024). 10.3390/vaccines12070771 PMC11281420 39066409

[16] Bet A., Maze E. A., Bansal A., Sterrett S., Gross A., Graff-Dubois S., Samri A., Guihot A., Katlama C., Theodorou I., Mesnard J. M., Moris A., Goepfert P. A., Cardinaud S., The HIV-1 antisense protein (ASP) induces CD8 T cell responses during chronic infection. Retrovirology 12, 15 (2015). 25809376 10.1186/s12977-015-0135-y PMC4335690

[17] Affram Y., Zapata J. C., Gholizadeh Z., Tolbert W. D., Zhou W., Iglesias-Ussel M. D., Pazgier M., Ray K., Latinovic O. S., Romerio F., The HIV-1 Antisense Protein ASP Is a Transmembrane Protein of the Cell Surface and an Integral Protein of the Viral Envelope. J Virol 93, (2019). 10.1128/JVI.00574-19 PMC6803264 31434734

[18] Kobayashi-Ishihara M., Terahara K., Martinez J. P., Yamagishi M., Iwabuchi R., Brander C., Ato M., Watanabe T., Meyerhans A., Tsunetsugu-Yokota Y., HIV LTR-Driven Antisense RNA by Itself Has Regulatory Function and May Curtail Virus Reactivation From Latency. Front Microbiol 9, 1066 (2018). 29887842 10.3389/fmicb.2018.01066 PMC5980963

[19] Zapata J. C., Campilongo F., Barclay R. A., DeMarino C., Iglesias-Ussel M. D., Kashanchi F., Romerio F., The Human Immunodeficiency Virus 1 ASP RNA promotes viral latency by recruiting the Polycomb Repressor Complex 2 and promoting nucleosome assembly. Virology 506, 34–44 (2017). 28340355 10.1016/j.virol.2017.03.002 PMC5505171

[20] Mancarella A., Procopio F. A., Achsel T., Crignis E. D., Foley B. T., Corradin G., Bagni C., Pantaleo G., Graziosi C., Detection of antisense protein (ASP) RNA transcripts in individuals infected with human immunodeficiency virus type 1 (HIV-1). Journal of General Virology, 863–876 (2019). 30896385 10.1099/jgv.0.001244

[21] McManus W. R., Bale M. J., Spindler J., Wiegand A., Musick A., Patro S. C., Sobolewski M. D., Musick V. K., Anderson E. M., Cyktor J. C., Halvas E. K., Shao W., Wells D., Wu X., Keele B. F., Milush J. M., Hoh R., Mellors J. W., Hughes S. H., Deeks S. G., Coffin J. M., Kearney M. F., HIV-1 in lymph nodes is maintained by cellular proliferation during antiretroviral therapy. J Clin Invest 129, 4629–4642 (2019). 31361603 10.1172/JCI126714 PMC6819093

[22] Wiegand A., Spindler J., Hong F. F., Shao W., Cyktor J. C., Cillo A. R., Halvas E. K., Coffin J. M., Mellors J. W., Kearney M. F., Single-cell analysis of HIV-1 transcriptional activity reveals expression of proviruses in expanded clones during ART. Proc Natl Acad Sci U S A 114, E3659–e3668 (2017). 28416661 10.1073/pnas.1617961114 PMC5422779

[23] Capoferri A. A., Wiegand A., Hong F., Jacobs J. L., Spindler J., Musick A., Bale M. J., Shao W., Sobolewski M. D., Cillo A. R., Luke B. T., Fennessey C. M., Gorelick R. J., Hoh R., Halvas E. K., Deeks S. G., Coffin J. M., Mellors J. W., Kearney M. F., HIV-1 control in vivo is related to the number but not the fraction of infected cells with viral unspliced RNA. Proc Natl Acad Sci U S A 121, e2405210121 (2024). 39190360 10.1073/pnas.2405210121 PMC11388345

[24] Hong F., Aga E., Cillo A. R., Yates A. L., Besson G., Fyne E., Koontz D. L., Jennings C., Zheng L., Mellors J. W., Novel Assays for Measurement of Total Cell-Associated HIV-1 DNA and RNA. J Clin Microbiol 54, 902–911 (2016). 26763968 10.1128/JCM.02904-15 PMC4809955

[25] Grubbs F. E., Sample Criteria for Testing Outlying Observations. The Annals of Mathematical Statistics 21, 27–58 (1950).

[26] Rose P. P., Korber B. T., Detecting hypermutations in viral sequences with an emphasis on G --> A hypermutation. Bioinformatics 16, 400–401 (2000). 10869039 10.1093/bioinformatics/16.4.400

[27] Sanders-Buell E., Salminen M., McCutchan F., Sequencing primers for HIV-1. Human retroviruses and AIDS, (1995).

[28] Swanson P., Devare S. G., Hackett J. Jr., Molecular characterization of 39 HIV isolates representing group M (subtypes A-G) and group O: sequence analysis of gag p24, pol integrase, and env gp41. AIDS Res Hum Retroviruses 19, 625–629 (2003). 12921095 10.1089/088922203322231003

[29] Ma G., Yasunaga J. I., Shimura K., Takemoto K., Watanabe M., Amano M., Nakata H., Liu B., Zuo X., Matsuoka M., Human retroviral antisense mRNAs are retained in the nuclei of infected cells for viral persistence. Proc Natl Acad Sci U S A 118, (2021). 10.1073/pnas.2014783118 PMC8092383 33875584

[30] Saayman S., Ackley A., Turner A. W., Famiglietti M., Bosque A., Clemson M., Planelles V., Morris K. V., An HIV-encoded antisense long noncoding RNA epigenetically regulates viral transcription. Mol Ther 22, 1164–1175 (2014). 24576854 10.1038/mt.2014.29 PMC4048891

[31] Djebali S., Davis C. A., Merkel A., Dobin A., Lassmann T., Mortazavi A., Tanzer A., Lagarde J., Lin W., Schlesinger F., Xue C., Marinov G. K., Khatun J., Williams B. A., Zaleski C., Rozowsky J., Röder M., Kokocinski F., Abdelhamid R. F., Alioto T., Antoshechkin I., Baer M. T., Bar N. S., Batut P., Bell K., Bell I., Chakrabortty S., Chen X., Chrast J., Curado J., Derrien T., Drenkow J., Dumais E., Dumais J., Duttagupta R., Falconnet E., Fastuca M., Fejes-Toth K., Ferreira P., Foissac S., Fullwood M. J., Gao H., Gonzalez D., Gordon A., Gunawardena H., Howald C., Jha S., Johnson R., Kapranov P., King B., Kingswood C., Luo O. J., Park E., Persaud K., Preall J. B., Ribeca P., Risk B., Robyr D., Sammeth M., Schaffer L., See L. H., Shahab A., Skancke J., Suzuki A. M., Takahashi H., Tilgner H., Trout D., Walters N., Wang H., Wrobel J., Yu Y., Ruan X., Hayashizaki Y., Harrow J., Gerstein M., Hubbard T., Reymond A., Antonarakis S. E., Hannon G., Giddings M. C., Ruan Y., Wold B., Carninci P., Guigó R., Gingeras T. R., Landscape of transcription in human cells. Nature 489, 101–108 (2012). 22955620 10.1038/nature11233 PMC3684276

[32] Battle A., Brown C. D., Engelhardt B. E., Montgomery S. B., Genetic effects on gene expression across human tissues. Nature 550, 204–213 (2017). 29022597 10.1038/nature24277 PMC5776756

[33] Forrest A. R. R., Kawaji H., Rehli M., Kenneth Baillie J., de Hoon M. J. L., Haberle V., Lassmann T., Kulakovskiy I. V., Lizio M., Itoh M., Andersson R., Mungall C. J., Meehan T. F., Schmeier S., Bertin N., Jørgensen M., Dimont E., Arner E., Schmidl C., Schaefer U., Medvedeva Y. A., Plessy C., Vitezic M., Severin J., Semple C. A., Ishizu Y., Young R. S., Francescatto M., Alam I., Albanese D., Altschuler G. M., Arakawa T., Archer J. A. C., Arner P., Babina M., Rennie S., Balwierz P. J., Beckhouse A. G., Pradhan-Bhatt S., Blake J. A., Blumenthal A., Bodega B., Bonetti A., Briggs J., Brombacher F., Maxwell Burroughs A., Califano A., Cannistraci C. V., Carbajo D., Chen Y., Chierici M., Ciani Y., Clevers H. C., Dalla E., Davis C. A., Detmar M., Diehl A. D., Dohi T., Drabløs F., Edge A. S. B., Edinger M., Ekwall K., Endoh M., Enomoto H., Fagiolini M., Fairbairn L., Fang H., Farach-Carson M. C., Faulkner G. J., Favorov A. V., Fisher M. E., Frith M. C., Fujita R., Fukuda S., Furlanello C., Furuno M., Furusawa J.-i., Geijtenbeek T. B., Gibson A. P., Gingeras T., Goldowitz D., Gough J., Guhl S., Guler R., Gustincich S., Ha T. J., Hamaguchi M., Hara M., Harbers M., Harshbarger J., Hasegawa A., Hasegawa Y., Hashimoto T., Herlyn M., Hitchens K. J., Ho Sui S. J., Hofmann O. M., Hoof I., Hori F., Huminiecki L., Iida K., Ikawa T., Jankovic B. R., Jia H., Joshi A., Jurman G., Kaczkowski B., Kai C., Kaida K., Kaiho A., Kajiyama K., Kanamori-Katayama M., Kasianov A. S., Kasukawa T., Katayama S., Kato S., Kawaguchi S., Kawamoto H., Kawamura Y. I., Kawashima T., Kempfle J. S., Kenna T. J., Kere J., Khachigian L. M., Kitamura T., Peter Klinken S., Knox A. J., Kojima M., Kojima S., Kondo N., Koseki H., Koyasu S., Krampitz S., Kubosaki A., Kwon A. T., Laros J. F. J., Lee W., Lennartsson A., Li K., Lilje B., Lipovich L., Mackay-sim A., Manabe R.-i., Mar J. C., Marchand B., Mathelier A., Mejhert N., Meynert A., Mizuno Y., de Lima Morais D. A., Morikawa H., Morimoto M., Moro K., Motakis E., Motohashi H., Mummery C. L., Murata M., Nagao-Sato S., Nakachi Y., Nakahara F., Nakamura T., Nakamura Y., Nakazato K., van Nimwegen E., Ninomiya N., Nishiyori H., Noma S., Nozaki T., Ogishima S., Ohkura N., Ohmiya H., Ohno H., Ohshima M., Okada-Hatakeyama M., Okazaki Y., Orlando V., Ovchinnikov D. A., Pain A., Passier R., Patrikakis M., Persson H., Piazza S., Prendergast J. G. D., Rackham O. J. L., Ramilowski J. A., Rashid M., Ravasi T., Rizzu P., Roncador M., Roy S., Rye M. B., Saijyo E., Sajantila A., Saka A., Sakaguchi S., Sakai M., Sato H., Satoh H., Savvi S., Saxena A., Schneider C., Schultes E. A., Schulze-Tanzil G. G., Schwegmann A., Sengstag T., Sheng G., Shimoji H., Shimoni Y., Shin J. W., Simon C., Sugiyama D., Sugiyama T., Suzuki M., Suzuki N., Swoboda R. K., ‘t Hoen P. A. C., Tagami M., Takahashi N., Takai J., Tanaka H., Tatsukawa H., Tatum Z., Thompson M., Toyoda H., Toyoda T., Valen E., van de Wetering M., van den Berg L. M., Verardo R., Vijayan D., Vorontsov I. E., Wasserman W. W., Watanabe S., Wells C. A., Winteringham L. N., Wolvetang E., Wood E. J., Yamaguchi Y., Yamamoto M., Yoneda M., Yonekura Y., Yoshida S., Zabierowski S. E., Zhang P. G., Zhao X., Zucchelli S., Summers K. M., Suzuki H., Daub C. O., Kawai J., Heutink P., Hide W., Freeman T. C., Lenhard B., Bajic V. B., Taylor M. S., Makeev V. J., Sandelin A., Hume D. A., Carninci P., Hayashizaki Y., The F. C., the R. P., Clst A promoter-level mammalian expression atlas. Nature 507, 462–470 (2014). 24670764 10.1038/nature13182 PMC4529748

[34] Ramilowski J. A., Yip C. W., Agrawal S., Chang J. C., Ciani Y., Kulakovskiy I. V., Mendez M., Ooi J. L. C., Ouyang J. F., Parkinson N., Petri A., Roos L., Severin J., Yasuzawa K., Abugessaisa I., Akalin A., Antonov I. V., Arner E., Bonetti A., Bono H., Borsari B., Brombacher F., Cameron C. J., Cannistraci C. V., Cardenas R., Cardon M., Chang H., Dostie J., Ducoli L., Favorov A., Fort A., Garrido D., Gil N., Gimenez J., Guler R., Handoko L., Harshbarger J., Hasegawa A., Hasegawa Y., Hashimoto K., Hayatsu N., Heutink P., Hirose T., Imada E. L., Itoh M., Kaczkowski B., Kanhere A., Kawabata E., Kawaji H., Kawashima T., Kelly S. T., Kojima M., Kondo N., Koseki H., Kouno T., Kratz A., Kurowska-Stolarska M., Kwon A. T. J., Leek J., Lennartsson A., Lizio M., López-Redondo F., Luginbühl J., Maeda S., Makeev V. J., Marchionni L., Medvedeva Y. A., Minoda A., Müller F., Muñoz-Aguirre M., Murata M., Nishiyori H., Nitta K. R., Noguchi S., Noro Y., Nurtdinov R., Okazaki Y., Orlando V., Paquette D., Parr C. J. C., Rackham O. J. L., Rizzu P., Sánchez Martinez D. F., Sandelin A., Sanjana P., Semple C. A. M., Shibayama Y., Sivaraman D. M., Suzuki T., Szumowski S. C., Tagami M., Taylor M. S., Terao C., Thodberg M., Thongjuea S., Tripathi V., Ulitsky I., Verardo R., Vorontsov I. E., Yamamoto C., Young R. S., Baillie J. K., Forrest A. R. R., Guigó R., Hoffman M. M., Hon C. C., Kasukawa T., Kauppinen S., Kere J., Lenhard B., Schneider C., Suzuki H., Yagi K., de Hoon M. J. L., Shin J. W., Carninci P., Functional annotation of human long noncoding RNAs via molecular phenotyping. Genome Res 30, 1060–1072 (2020). 32718982 10.1101/gr.254219.119 PMC7397864

[35] Hon C.-C., Ramilowski J. A., Harshbarger J., Bertin N., Rackham O. J. L., Gough J., Denisenko E., Schmeier S., Poulsen T. M., Severin J., Lizio M., Kawaji H., Kasukawa T., Itoh M., Burroughs A. M., Noma S., Djebali S., Alam T., Medvedeva Y. A., Testa A. C., Lipovich L., Yip C.-W., Abugessaisa I., Mendez M., Hasegawa A., Tang D., Lassmann T., Heutink P., Babina M., Wells C. A., Kojima S., Nakamura Y., Suzuki H., Daub C. O., de Hoon M. J. L., Arner E., Hayashizaki Y., Carninci P., Forrest A. R. R., An atlas of human long non-coding RNAs with accurate 5′ ends. Nature 543, 199–204 (2017). 28241135 10.1038/nature21374 PMC6857182

[36] Seifuddin F., Singh K., Suresh A., Judy J. T., Chen Y.-C., Chaitankar V., Tunc I., Ruan X., Li P., Chen Y., Cao H., Lee R. S., Goes F. S., Zandi P. P., Jafri M. S., Pirooznia M., lncRNAKB, a knowledgebase of tissue-specific functional annotation and trait association of long noncoding RNA. Scientific Data 7, 326 (2020). 33020484 10.1038/s41597-020-00659-z PMC7536183

[37] Josefsson L., von Stockenstrom S., Faria N. R., Sinclair E., Bacchetti P., Killian M., Epling L., Tan A., Ho T., Lemey P., Shao W., Hunt P. W., Somsouk M., Wylie W., Douek D. C., Loeb L., Custer J., Hoh R., Poole L., Deeks S. G., Hecht F., Palmer S., The HIV-1 reservoir in eight patients on long-term suppressive antiretroviral therapy is stable with few genetic changes over time. Proc Natl Acad Sci U S A 110, E4987–4996 (2013). 24277811 10.1073/pnas.1308313110 PMC3870728

[38] Mancarella A., Procopio F. A., Achsel T., De Crignis E., Foley B. T., Corradin G., Bagni C., Pantaleo G., Graziosi C., Detection of antisense protein (ASP) RNA transcripts in individuals infected with human immunodeficiency virus type 1 (HIV-1). J Gen Virol 100, 863–876 (2019). 30896385 10.1099/jgv.0.001244

[39] Bruner K. M., Wang Z., Simonetti F. R., Bender A. M., Kwon K. J., Sengupta S., Fray E. J., Beg S. A., Antar A. A. R., Jenike K. M., Bertagnolli L. N., Capoferri A. A., Kufera J. T., Timmons A., Nobles C., Gregg J., Wada N., Ho Y. C., Zhang H., Margolick J. B., Blankson J. N., Deeks S. G., Bushman F. D., Siliciano J. D., Laird G. M., Siliciano R. F., A quantitative approach for measuring the reservoir of latent HIV-1 proviruses. Nature 566, 120–125 (2019). 30700913 10.1038/s41586-019-0898-8 PMC6447073

[40] Mansky L. M., Temin H. M., Lower in vivo mutation rate of human immunodeficiency virus type 1 than that predicted from the fidelity of purified reverse transcriptase. J Virol 69, 5087–5094 (1995). 7541846 10.1128/jvi.69.8.5087-5094.1995 PMC189326

