# Supplementary material for: In vivo detection of HIV-1 antisense transcripts in untreated and ART-treated individuals
Source: bioRxiv. 2025 Feb 25:2024.12.06.627170. Preprint. [Version 2] doi: 10.1101/2024.12.06.627170 (PMC11908204; doi:10.1101/2024.12.06.627170)
Supplement: Supplement 1 [file NIHPP2024.12.06.627170V2-supplement-1.pdf]

bioRxiv preprint doi: https://doi.org/10.1101/2024.12.06.627170; this version posted February 25, 2025. The copyright holder for this preprint (which was not certified by peer review) is the author/funder. This article is a US Government work. It is not subject to copyright under 17 USC 105 and is also made available for use under a CC0 license.

Supplementary Materials for

In vivo detection of HIV antisense transcripts in untreated and ART-treated individuals

Adam A. Capoferri[1]*†, Rachel Sklutuis[1]†, Toluleke O. Famuyiwa[1], Sachi Pathak[1], Rui Li[2], Jason W. Rausch[1], Brian T. Luke[3], Rebecca Hoh[4], Steven G. Deeks[4], John W. Mellors[5], John M. Coffin[6], Jennifer L. Groebner[1], Fabio Romerio[2], Mary F. Kearney[1*]

Corresponding author: kearneym@mail.nih.gov and adam.capoferri@nih.gov

The PDF file includes:

Materials and Methods
Figs. S1 to S4
Tables S1 to S6
References 37-40

bioRxiv preprint doi: https://doi.org/10.1101/2024.12.06.627170; this version posted February 25, 2025. The copyright holder for this preprint (which was not certified by peer review) is the author/funder. This article is a US Government work. It is not subject to copyright under 17 USC 105 and is also made available for use under a CC0 license.

Materials and Methods

Participant cohorts, sample collection, and study approval

PBMC were collected from PWH on ART who were enrolled at the University of California of San Francisco in the SCOPE trial (clinical trial # NCT00187512). PBMC were also collected from PWH who were not currently on ART and were enrolled at the University of Pittsburgh in the Optimization of Immunologic and Virologic Assays for HIV Trial (IRB# STUDY20040215). The studies were approved by the University of California San Francisco Institutional Review Board and the University of Pittsburgh Institutional Review Board. All donors provided written informed consent for HIV-1 quantification and sequencing. PBMC were separated using Ficoll, resuspended in FBS with 10% DMSO, and stored in liquid nitrogen (LN_2) until testing.

Overview of HIV-1 antisense transcript (AST) quantification and sequencing approaches

HIV-1 AST quantification and sequencing assays were performed by modifying our cell-associated RNA and DNA single-genome sequencing assay (CARD-SGS) (22, 23) (detailed methods below). Briefly, total nucleic acid was extracted from serially diluted aliquots of PBMC collected from PWH, the DNA was digested, and AST cDNA synthesized using a gene-specific primer in the opposite orientation of the *env* coding region (**Fig. S1B**). The cDNA from each aliquot was spread across 96-well PCR plates for amplification (~50bp) and probe detection to determine the dilution that yielded <30% positive PCR products, indicating that cDNA was primarily amplified from single molecules. By determining the endpoint dilution, we could quantify the number of AST in each aliquot and determine the fraction of the infected cells with AST by using the number of HIV-1 DNA molecules in replicate aliquots for the denominator. HIV-1 DNA was measured with the integrase cell-associated DNA (iCAD) assay (24). The number of HIV-1 DNA molecules is a surrogate for the number of infected cells since previous studies showed that most infected cells carry only a single provirus both before and during ART (37). For a subset of the samples, a 1.7-kb fragment of AST was also amplified at an endpoint and Sanger sequenced to assess the genetic diversity of the transcripts.

Overview of the development and optimization of AST quantification and sequencing

During the development and optimization of the HIV-1 AST quantification and sequencing assays, we controlled for endogenous priming during cDNA synthesis and contamination of HIV-1 DNA. We also determined the sensitivity and background of the AST assays. An overview of these developmental steps is provided here, but detailed protocols for each set of experiments are provided below.

1. To eliminate endogenous self-priming during AST cDNA synthesis (19, 38), we used a 5′ end exogenous oligo-tagged gene specific primer (**Table S1**). The tag generates cDNA carrying the

bioRxiv preprint doi: https://doi.org/10.1101/2024.12.06.627170; this version posted February 25, 2025. The copyright holder for this preprint (which was not certified by peer review) is the author/funder. This article is a US Government work. It is not subject to copyright under 17 USC 105 and is also made available for use under a CC0 license.

exogenous tag sequence to function as the target for the forward primer during first-round PCR amplification; thus, allowing for specific amplification of cDNA molecules containing the exogenous oligo-tag sequence, rather than endogenous self-primed templates.

2. To ensure complete DNA digestion, we used various amounts of the ACH-2 infected cell line (~1 provirus/cell), spiked into 1x10^5 or 1x10^6 uninfected CEM cells (**Fig. S2**). Nucleic acid was extracted, DNase treated, and "cDNA synthesized" without reverse transcriptase. Nested PCR was performed to determine the maximum number of cells that can be assayed to ensure that HIV DNA digestion was complete. We found complete digestion of viral DNA at 100 infected cells per aliquot, in agreement with prior findings with the CARD-SGS assay where <300 infected cells per aliquot was the upper limit (*22*).
3. To optimize the AST assay, we used *in vitro* transcribed AST generated with a pMiniT-AST vector containing a T7 promoter (**Fig. S3**). The concentration of AST was measured by spectrophotometry and 10^4 copies were spiked into nucleic acid extracted from 1x10^5 uninfected CEM cells. The number of positives detected in the assay was compared to the known number of transcripts. The optimized AST detection assay had a sensitivity of ~50% (average of 5,589 copies detected), which could be a consequence of including the exogenous oligo-tag on the cDNA primer reducing cDNA synthesis efficiency or loss of RNA template from the purification steps of the assay.
4. After optimizing the assay using *in vitro* transcribed AST, we tested the assay on 100 ACH-2 cells spiked into 10^5 uninfected CEM cells (**Fig. S4**). Using the AST assay (43bp amplicon) at endpoint with probe detection, we found a median of 41 AST molecules per 100 infected ACH-2 cells [IQR 25-69] (n=16 replicates). We detected 13 AST /100 infected ACH-2 cells when amplifying and sequencing a 1.7-kb fragment of anti-*env*. The less frequent detection of the 1.7-kb amplicon is not unexpected compared to the 43bp amplicon.
5. To determine the cut-off for the digital (probe detection at an endpoint) AST assay, we performed replicates of "no reverse-transcriptase (RT)" controls on 100 ACH-2 cells spiked into 10^5 uninfected CEM cells. We found 4 potential false positive signals in 650 infected cells assayed when RT was not included in the cDNA reaction, making our assay cut-off 0.6 AST/100 infected cells. We also tested 5x10^5 uninfected CEM cells spread across 182 PCR wells and detected 1 false positive, making the false-positive assay background <0.0002/100 cells assayed. To overcome the background, we limited each digital assay to <100 infected cells and included equal numbers of "no RT" controls on each PCR plate.

Cell lines, maintenance, and storage

bioRxiv preprint doi: https://doi.org/10.1101/2024.12.06.627170; this version posted February 25, 2025. The copyright holder for this preprint (which was not certified by peer review) is the author/funder. This article is a US Government work. It is not subject to copyright under 17 USC 105 and is also made available for use under a CC0 license.

Cell lines used for assay development were uninfected CEM/C1 cells (American Type Culture Collection, #CRL-2265) and HIV-1 infected ACH-2 cells (HIV Reagent Program, #ARP-349). ACH-2 cells are A3.01 cells infected with a single integrated provirus (HIV-1_{LAI}) with a defect in TAR (C37T). Complete Medium was prepared as RPMI 1640 supplemented with 1% Penicillin-Streptomycin-Glutamine (Gibco, #10378016) and 10% heat-inactivated fetal bovine serum (HI-FBS). Cell Freeze Medium was prepared as RPMI 1640 supplemented with 10% HI-FBS (CEM/C1 cells) or 10-40% HI-FBS (ACH-2 cells), and 10% DMSO.

Cells were maintained in Complete Medium (as described above) and seeded in 2x vented T-25 flasks (1:3 and 2:3 from vial contents) with a total of 6mL in each flask. Cells were incubated overnight at 37°C, 5% CO_2 with flasks upright. The following day, the medium was aspirated without disturbing the cells and were replenished with warmed Complete Medium. The cells were subcultured and transferred to a 15mL conical tube for centrifugation at 150x*g* for 10 minutes, then media was aspirated. The cells were resuspended in 1mL Complete Medium and counted by hemocytometer at 1:10 and 1:100 dilutions. Viable cells were seeded at 2-4x10^5 in new T-25 flasks with 6mL of warmed Complete Medium. If needed, cells were expanded in T-75 flasks (seeding at 4-6x10^5) with medium volume between 10-20mL. The cells were split every 2-3 days as needed.

The cells were prepared in Cell Freeze Medium at a minimum of 1x10^6 cells/mL by gentle pipetting of cells and aliquoted into labeled cryotubes. They were placed in a freezing container with 100% isopropanol in outer container to freeze cells at -80°C overnight. The following day, the cells were transferred to LN_2 storage.

Sample Preparation

To thaw viably frozen cells, RPMI 1640 was warmed to 37°C and added dropwise to thaw PBMC for nucleic acid extraction. Each viably frozen cell vial was warmed for ~2 minutes at 37°C prior to RPMI being added dropwise to the vial. Thawed donor PBMC were aliquoted and centrifuged at 500×*g* for 5 minutes. Supernatant was removed and the pelleted cells were used immediately for downstream assays or were frozen on dry ice and stored back in LN_2 until ready for use.

Generation of control RNA

RNA controls were generated to determine the efficiency of the HIV-1 AST cDNA synthesis and PCR amplification. AST (*anti-env*) were amplified from a pUC57-AST plasmid obtained from Fabio Romerio (Department of Molecular and Comparative Pathobiology, The Johns Hopkins University School of Medicine, Baltimore, MD) using M13 Fwd and M13 Rev primers (**Table S2**). Using Platinum II Taq (ThermoFisher, #14966025), 50µL PCR reactions were carried out with 50ng of plasmid DNA and 10µL

bioRxiv preprint doi: https://doi.org/10.1101/2024.12.06.627170; this version posted February 25, 2025. The copyright holder for this preprint (which was not certified by peer review) is the author/funder. This article is a US Government work. It is not subject to copyright under 17 USC 105 and is also made available for use under a CC0 license.

of 5X Platinum II PCR Buffer, 1µL of 10mM dNTPs, 1µL of each 10µM primer, 0.4µL of Platinum II Taq polymerase, and molecular-grade water to bring to 50µL. PCR cycling was performed as follows: 94°C for 2 minutes and 45 cycles of 94°C for 15 seconds, 60°C for 15 seconds, and 68°C for 45 seconds, followed by a final extension at 68°C for 1 minute. The PCR product was then purified using a QIAquick PCR Purification Kit (QIAGEN, #28104). The PCR product was cloned using NEB® PCR Cloning Kit (New England Biolabs, #E1202S) where 80ng of the purified PCR product was ligated into a linearized pMiniT 2.0 vector and transformed into the NEB® stable competent 10-beta *E. coli* following manufacturer's instructions (New England Biolabs, #C3019H). Transformed colonies were picked, grown overnight according to manufacturer's instructions, and miniprepped using the QuickLyse Miniprep Kit (QIAGEN, #27406). Plasmids were screened for the insert by selective PCR screening using Cloning Analysis Fwd and Rev primers (**Table S2**) with PCR cycling performed as follows: 94°C for 2 minutes and 45 cycles of 94°C for 15 seconds, 60°C for 15 seconds, and 68°C for 45 seconds, followed by a final extension at 68°C for 1 minute. The plasmid was then Sanger sequenced to confirm orientation and sequence (**Table S2**).

To generate control transcripts, 1µg of the AST clone (mentioned above) was digested by restriction enzyme PmeI (New England Biolabs, #R0560S) for AST (*anti-env*) or ZraI (New England Biolabs, #R0659S) for sense *env* transcripts following manufacturer's instructions. Digested DNA was then purified using the QIAquick PCR Purification Kit (QIAGEN, #28104) and concentrated using ethanol precipitation (described below). RNA was transcribed using HiScribe T7 Quick High Yield RNA Synthesis Kit (New England Biolabs, #E2050S) for AST or the HiScribe SP6 Quick High Yield RNA Synthesis Kit (New England Biolabs, #E2070S) for sense *env* transcripts. Transcribed RNA was purified using a Monarch RNA Cleanup Kit (New England Biolabs, #T2030) and quantified by Nanodrop spectrophotometer. The RNA was diluted to $1x10^6$ copies/mL with 5mM Tris-HCl pH 8.0 and stored at -80°C for temporary storage and LN_2 for longer storage to help avoid degradation of RNA.

To assess the completeness of digestion of plasmid DNA, approximately 10^4 copies of the transcripts were spiked into extracted nucleic acid from 10^5 CEM cells and DNase I treated (described below). The control transcripts were used for cDNA synthesis using random hexamer primers (ThermoFisher, #SO142) in the following reaction: 2.5µL 10mM dNTPs and 2.5µL 2µM random hexamer primer were added to each 20µL RNA sample. The RNA was denatured at 65°C for 10 minutes, then immediately cooled at -20°C for 1 minute. Next, 25µL of SuperScript III Reverse Transcriptase (Invitrogen, #18080-044) master mix was added to the denatured RNA. Master mix was made by combining 10µL 5X first strand buffer, 1µL 0.1M DTT, 12µL RNase-free water, 1µL 40U/µL RNaseOUT recombinant ribonuclease inhibitor (Invitrogen, #10777-019), and 1µL 200U/µL SuperScript III Reverse Transcriptase. cDNA was synthesized at 25°C for 5 minutes, 55°C for 1 hour, 70°C for 15 minutes, then cooled to 4°C. To remove hybridized RNA, 1µL RNase H (5 units) (New England Biolabs, #M0297S) was added and

bioRxiv preprint doi: https://doi.org/10.1101/2024.12.06.627170; this version posted February 25, 2025. The copyright holder for this preprint (which was not certified by peer review) is the author/funder. This article is a US Government work. It is not subject to copyright under 17 USC 105 and is also made available for use under a CC0 license.

incubated at 37°C for 20 minutes followed by an enzyme heat-inactivation at 75°C for 10 minutes, and the cDNA was cooled to 4°C and used immediately or stored at -80°C.

DNA and RNA Extraction

Total nucleic acid was extracted from endpoint diluted aliquots of HIV-infected cells with AST RNA, determined by performing serial dilutions of PBMC until 96-well PCR plates yielded <30% AST positives. Nucleic acid was extracted by adding 100μL 3M guanidine HCl solution (18.75mL 8M guanidinium HCl, 2.5mL 1M Tris-HCl pH 8.0, 0.5mL 100mM $CaCl_2$) and 5μL 20mg/mL Proteinase K to a cell pellet, vortexed, and incubated at 42°C heat block for 1 hour. Addition of 400μL 6M guanidine isothiocyanate solution (25mL 6M guanidinium isothiocyanate, 14mL 1M Tris-HCl pH 8.0, 53μL EDTA pH 8.0) and 6μL 20mg/mL glycogen to lysed cell pellet was vortexed, and incubated at 42°C heat block for 10 minutes. Five hundred microliters of 100% isopropanol was added with mixing, followed by centrifugation at 21,000x*g* for 10 minutes at room temperature to precipitate nucleic acids. Supernatant was removed without disturbing the pellet and washed with 750μL 70% ethanol. Precipitated nucleic acid was air-dried. For DNA, the pellet was resuspended in 100μL 5mM Tris-HCl pH 8.0 and ready for use. For RNA, the DNA was digested by either (i) DNase I (Roche, #04716728001) or (ii) ezDNase (ThermoFisher, #11766051):

(i) DNase I: The air-dried extracted nucleic acid was resuspended in 38μL DNase buffer and 2μL of 10units/μL DNase I and incubated for 20 minutes in a 37°C water bath. After incubation, 200μL of 6M GuSCN (Sigma, #50983) was added and mixed well, followed by the addition of 250μL of 100% isopropanol. Sample was vortexed for 10 seconds, then centrifuged at 21,000×*g* for 10 minutes to pellet nucleic acid. Supernatant was removed and pellet was washed with 1mL 70% ethanol, followed by additional centrifugation at 21,000×*g* for 10 minutes. Finally, the DNA-digested RNA pellet was air-dried, resuspended in 20μL of 5 mM Tris-HCl pH 8.0, and used immediately for cDNA synthesis. Alternatively, RNA could be temporarily stored in 70% ethanol at -80°C.

(ii) ezDNase: The air-dried extracted nucleic acid was resuspended in 32μL 5mM Tris-HCl pH 8.0. Sample was split in equal volumes to control for successful DNA digestion. To ensure successful DNA digestion, manufacturer's instructions were followed, with volumes of reagents doubled and maximum incubations used. Briefly, 2μL 10X ezDNase Buffer and 2μL ezDNase were added to each sample and incubated at 37°C for 5 minutes. To inactivate the ezDNase, the mixture was incubated at 55°C for 5 minutes in the presence of 2μL 0.1M DTT. Incubations were performed in a thermocycler. Samples were immediately cooled at -20°C and brought up to room temperature before directly moving into cDNA synthesis.

bioRxiv preprint doi: https://doi.org/10.1101/2024.12.06.627170; this version posted February 25, 2025. The copyright holder for this preprint (which was not certified by peer review) is the author/funder. This article is a US Government work. It is not subject to copyright under 17 USC 105 and is also made available for use under a CC0 license.

cDNA Synthesis

The cDNA was synthesized by adding 2.5µL 10mM dNTPs and 2.5µL 2µM gene-specific tagged cDNA primer (AST cDNA, **Table S1**) to each sample. The RNA was denatured at 85°C for 10 minutes, then immediately cooled at -20°C for 1 minute. Next, 25µL SuperScript III Reverse Transcriptase (Invitrogen, #18080-044) master mix was added to the denatured RNA. Master mix was 10µL 5X first strand buffer, 0.5µL 0.1M DTT, 13.5µL RNase-free water, 0.5µL 40 U/µL RNaseOUT recombinant ribonuclease inhibitor (Invitrogen, #10777-019), and 1µL 200 U/µL SuperScript III Reverse Transcriptase. cDNA was synthesized at 45°C for 1 hour, then cooled to 4°C. To remove hybridized RNA, 1µL RNase H (5 units) (New England Biolabs, #M0297S) was added and incubated at 37°C for 20 minutes followed by an enzyme heat-inactivation at 75°C for 10 minutes. The cDNA was cooled to 4°C and was transferred to a low bind Eppendorf tube and 0.1 vol 3M Sodium Acetate pH 5.5 (Invitrogen, #AM9740) and 1µL 20mg/mL glycogen (Roche, #1090139300) were added. After mixing, 3 volumes 95% ethanol was added, and the entire mixture was vortexed for 10 seconds and incubated overnight at -20°C. The next day, the precipitated cDNA was centrifuged at 21,000x*g* for 20 minutes at room temperature, and supernatant was removed. The pellet was washed with 600µL 70% ethanol, pulse vortexed, and centrifuged for 15 minutes at 21,000x*g* at room temperature. Supernatant was removed and cDNA pellet was air dried and then resuspended in 20µL or 50µL (for digital PCR quantitation) in 5mM Tris-HCl pH 8.0. The cDNA was then used immediately or stored at -80°C.

Quantification of HIV-1 infected cells

The number of HIV infected cells was estimated from the levels of HIV DNA in PBMC. For donors on ART, HIV-1 DNA levels were previously measured using the integrase cell-associated single-copy DNA (iCAD) assay (primers in **Table S3**) (*21*, *24*). We also measured HIV-1 DNA levels using a modified primer probe set in the Rev Response Element (RRE) adapted from the Intact Proviral DNA Assay (*39*) (**Table S4**). Digital PCR was performed using Lightcycler 480 Probes Master hot-start reaction mix (Roche, #4707494001) in 20µL volumes per reaction. A 2mL master mix was prepared by combining 1mL 2X Lightcycler 480 Probes Master hot-start reaction mix, 12µL 100µM RRE Fwd primer, 12µL 100µM RRE Rev, 2µL 100µM RRE probe, 774µL RNase-free water, and 200µL diluted DNA (DNA was diluted up to 200µL with 5mM Tris-HCl pH 8.0). Digital PCR was performed on the Roche LightCycler480 at a denaturation temperature of 95ºC for 10 minutes and 55 cycles (95ºC for 15 seconds and 60ºC for 1 minute). Equal numbers of "no RT" controls and experimental wells were included on each plate to ensure complete HIV-1 DNA digestion. Two negative (no template, 5mM Tris-HCl pH 8.0) and two positive (100 ACH-2 in a background $1x10^5$ CEM cells of DNA) controls were also included on each PCR plate. The number of

bioRxiv preprint doi: https://doi.org/10.1101/2024.12.06.627170; this version posted February 25, 2025. The copyright holder for this preprint (which was not certified by peer review) is the author/funder. This article is a US Government work. It is not subject to copyright under 17 USC 105 and is also made available for use under a CC0 license.

positive wells was used to calculate the number of infected cells per million PBMC in each of the donor samples.

Design of gene-specific primers and probes

Due to intra- and inter-host HIV-1 sequence variation, we extracted HIV-1 DNA from PBMC (as described above) and performed PCR at endpoint targeting *env* (primers in **Table S1**) and performed Sanger sequencing (primers in **Table S4**). The designed primers for were used accordingly (**Table S5**, donor-specific mutations are shown in bold red).

AST quantification by digital PCR

AST digital PCR was performed using Lightcycler 480 Probes Master hot-start reaction mix (Roche, #4707494001) in 20µL volumes per reaction. A master mix was prepared by combining 2X Lightcycler 480 Probes Master hot-start reaction mix, 100µM Fwd primer, 100µM Rev primer, 100µM AST probe, RNase-free water, and cDNA synthesized as described above. Digital PCR was performed on the Roche LightCycler480 at a denaturation temperature of 95ºC for 10 minutes and 55 cycles (95ºC for 15 seconds and 60ºC for 1 minute). Equal numbers of "no RT" controls and experimental wells were included on each plate to ensure complete HIV-1 DNA digestion. Two negative (no template, 5mM Tris-HCl pH 8.0) and two positive (100 ACH-2 in a background $1x10^5$ CEM cells of DNA) controls were also included on each PCR plate.

AST quantification and sequencing

AST quantification and sequencing was modified from the original CARD-SGS assay protocol as previously described (22, 23) and was used to sequence and quantify HIV-1 DNA and sense and antisense HIV RNA in *gag, pol,* and *env*. Briefly, cDNA or DNA was diluted to a near endpoint in a volume of 200µL and added to 800µL Platinum II Taq (ThermoFisher Scientific, #14966025) master mix containing 200µL 5X Platinum II PCR Buffer, 20µL 10mM dNTPs, 8µL primers (4µL 50µM Forward and 4µL 50µM Reverse primers, for respective region, **Table S1**), 564µL molecular-grade water, and 8µL Platinum II Taq polymerase. The total volume of cDNA and master mix was spread across a 96-well plate (10µL per well). PCR cycling was performed with respect to sub-genomic region (**Table S1**). Following the first round of PCR, each well was diluted with 50µL 5mM Tris-HCl pH 8.0. For nested PCR, 2µL diluted PCR 1 product was transferred from each well to a 96-well plate containing 8µL Platinum II Taq master mix (described above). Nested PCR was performed with respect to sub-genomic region with respective thermocycling conditions (**Table S1**). Nested PCR product was diluted with 50µL 5mM Tris-HCl pH 8.0. Positive wells

bioRxiv preprint doi: https://doi.org/10.1101/2024.12.06.627170; this version posted February 25, 2025. The copyright holder for this preprint (which was not certified by peer review) is the author/funder. This article is a US Government work. It is not subject to copyright under 17 USC 105 and is also made available for use under a CC0 license.

were identified by GelRed detection (Biotium, #41003) using UV imaging. Positive PCR reactions were sent for Sanger sequencing (**Table S4**).

The levels of HIV expression in single cells were determined by the number of identical sequences in each aliquot. Because the reverse transcription step is known to introduce errors at a rate of about 10^{-4} per sequenced nucleotide of viral cDNA sequences (*40*), RT-PCR variants that differed by a single nucleotide ('fuzz') from a group of 7 or more identical sequences within the same aliquot were, conservatively, considered to belong to the same rake of identical sequences. The R script for "defuzzing" is available at https://github.com/michaelbale/RStuff/blob/master/fPECS_fncs.R.

Sequence Analysis

Sequences were aligned with MAFFT v7.450 (FFT-NS-1 200PAM/k=2 algorithm) in Geneious Prime ® 2020.2.4. Minor adjustments were performed manually. P-distance neighbor joining trees were reconstructed using MEGA X and rooted on the consensus B HIV sequence. Population genetic diversity was calculated as average pairwise distance (APD) using MEGA 11. APOBEC3-F/G mediated hypermutation was predicted using the Los Alamos National Laboratory HIV Sequence database tool, Hypermut (https://www.hiv.lanl.gov/content/sequence/HYPERMUT/hypermut.html).

Data availability:

All sequence data are available on GenBank (in process of submission). Sequences from the donors on ART were previously published by McManus and colleagues and can be found in (*21*).

Statistical analyses

Mann-Whitney U-test, Kruskal-Wallis test, Spearman's correlation, Grubbs' Outlier test, and Shapiro-Wilk test for normality for informed Paired T-test were performed using Prism GraphPad 9.2. Spearman's correlation was selected due to the random variation in biological systems.

To test if there was a difference in the levels of AST pre- and post-ART interruption we used a binomial test. From the two sample donations pre-interruption, a total of 155 infected PBMC were assayed and 39 infected PBMC were found to contain AST (25%). Post-interruption, 133 infected PBMC were assayed and 65 infected PBMC contained AST (49%). A Binomial distribution was used to show that 65 out of 133 is significantly larger than 39 out of 155.

Statistical tests applied are indicated in the text and/or figures/table legends. Additional statistical analyses were performed using R (version 4.3.1).

bioRxiv preprint doi: https://doi.org/10.1101/2024.12.06.627170; this version posted February 25, 2025. The copyright holder for this preprint (which was not certified by peer review) is the author/funder. This article is a US Government work. It is not subject to copyright under 17 USC 105 and is also made available for use under a CC0 license.

Fig. S1

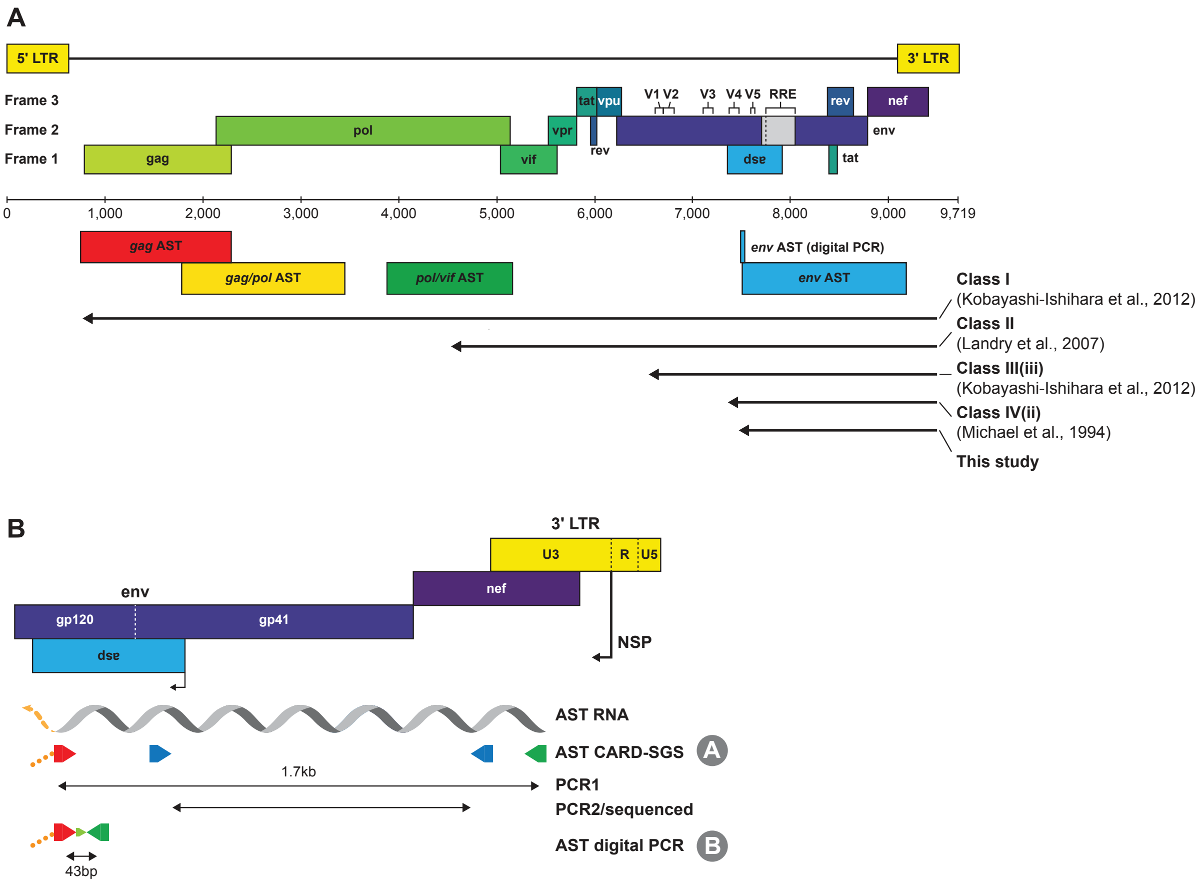

Fig. S1. Assays for measuring levels and sequencing AST *in vivo*. (**A**) Measuring AST along the HIV-1 genome. The antisense regions for single-genome sequencing of *gag, gag/pol*, *pol/vif*, and *env* are shown with the anti-*env* location for the digital PCR assay indicated as well. The genome map is relative to HXB2. Different length Classes of AST are defined for Class I (*12*), Class II (*11*), Class III(iii) (*12*), Class IV (*5*), and in our study. (**B**) A detailed view of the locations for the AST CARD-SGS assay (protocol A) and the digital PCR assay (protocol B). The negative sense promoter (nsp) for AST is shown at the 3′ LTR U3/R junction. The orange segment of the AST RNA indicates the potential for the transcript to be longer such as Class I & II. The tag for the primers is bold dashed orange that stems from the cDNA synthesis primer (red). Protocol A for AST CARD-SGS includes PCR1 with the forward primer to the tag to amplify a ~1.7-kb fragment from gp120 *env* to *nef*/U3, and a nested PCR before sequencing. Protocol B for AST digital PCR, uses the forward primer binding to the tag, and amplifies 43bp. The AST digital PCR assay amplification region overlaps the AST CARD-SGS assay region in *env*.

bioRxiv preprint doi: https://doi.org/10.1101/2024.12.06.627170; this version posted February 25, 2025. The copyright holder for this preprint (which was not certified by peer review) is the author/funder. This article is a US Government work. It is not subject to copyright under 17 USC 105 and is also made available for use under a CC0 license.

Fig. S2

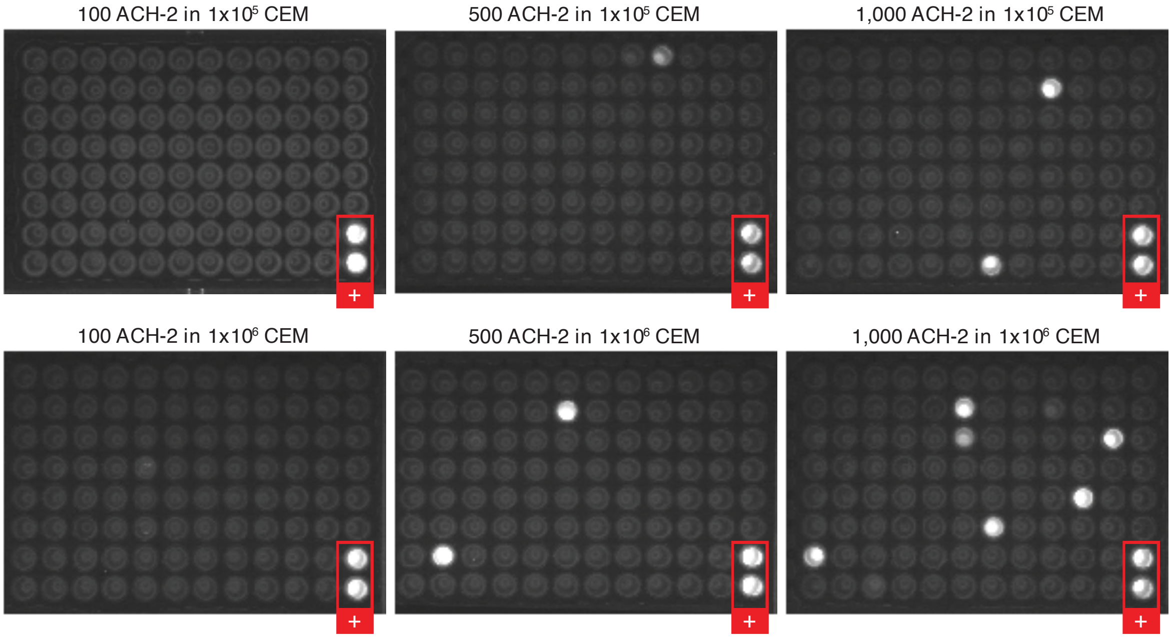

Fig. S2. Development of the Antisense Transcripts Cell-Associated RNA and DNA Single Genome Sequencing (AST CARD-SGS) assay – control for DNA digestion. cDNA reactions were performed with the exogenous oligo-tagged RT primer but excluding the reverse transcriptase enzyme, SuperScript III. The reaction was then spread across a 96-well plate and nested PCR was completed. Each plate included positive control wells (red box) that contained ACH-2 DNA.

bioRxiv preprint doi: https://doi.org/10.1101/2024.12.06.627170; this version posted February 25, 2025. The copyright holder for this preprint (which was not certified by peer review) is the author/funder. This article is a US Government work. It is not subject to copyright under 17 USC 105 and is also made available for use under a CC0 license.

Fig. S3

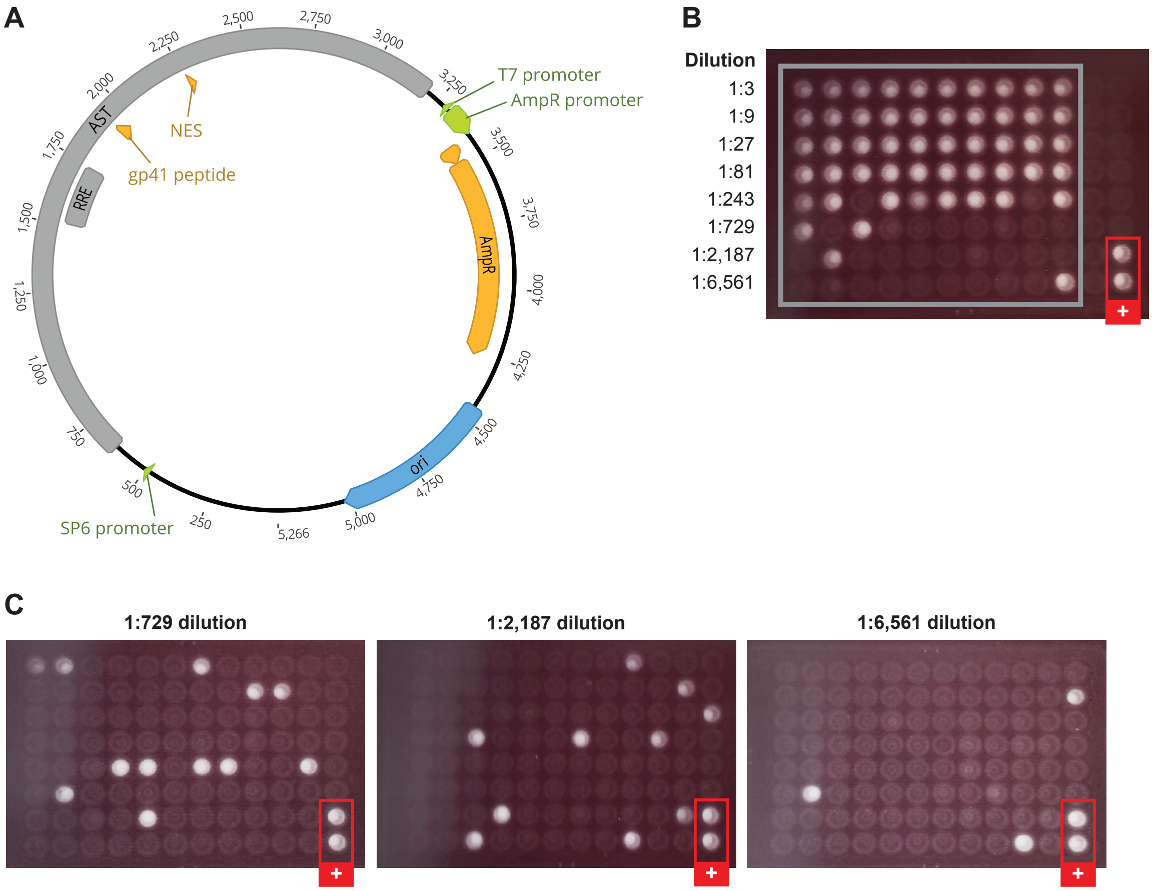

D

Dilution	Positive wells in 50uL	Copies in 150uL	Total copies	Average
1:729	12	36	2,916	5,589
1:2,187	10	3	7,290	
1:6,561	3	9	6,561	

Fig. S3. Determining the sensitivity of the AST CARD-SGS. (**A**) Plasmid used for *in vitro* expression of AST (pMiniT Expression Vector). (**B**) PCR replicates of serially diluted *in vitro* transcribed, then reverse transcribed AST for quantification and endpoint determination (10^4 copies of AST RNA, determined by spectrophotography, were spiked into nucleic acid extracted from 10^5 uninfected CEM cells (to mimic the 1/1,000 infected cells:uninfected cells in PWH). 2µL of each dilution was added to each PCR reaction well. (**C**) Detection plates of 50µL of select dilutions determined at endpoint. Detection plates were performed with 150µL as well (not shown, but results provided in **Fig. S3D**). (**D**) Average total number of detected AST copies based on the number of positives in the detection plates. Of 10^4 AST RNA copies, 5,589 were recovered, showing that the sensitivity of our methods for AST detection is about 50%.

bioRxiv preprint doi: https://doi.org/10.1101/2024.12.06.627170; this version posted February 25, 2025. The copyright holder for this preprint (which was not certified by peer review) is the author/funder. This article is a US Government work. It is not subject to copyright under 17 USC 105 and is also made available for use under a CC0 license.

Fig. S4

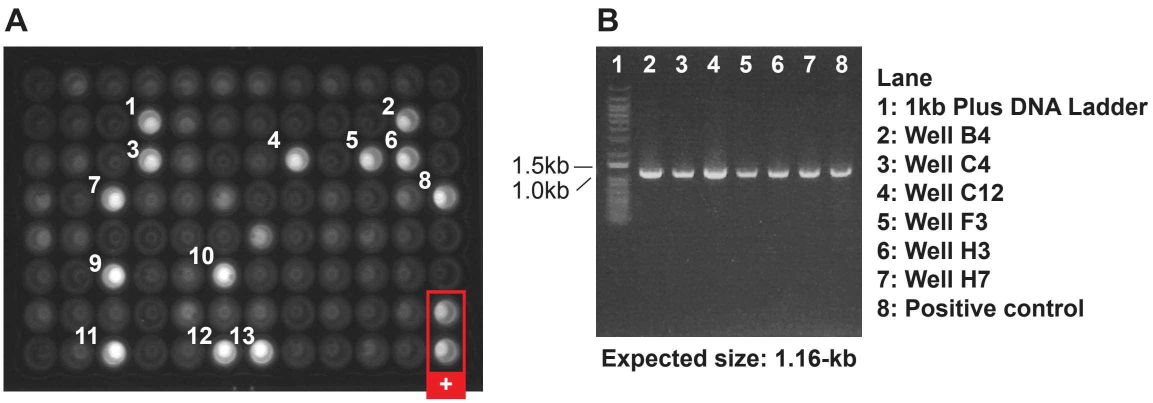

Fig. S4. Optimized AST CARD-SGS Assay. (**A**) The *env* AST CARD-SGS assay was applied to an aliquot of 100 ACH-2 cells in a background of $1x10^5$ CEM cells. (**B**) Select positive amplicons from the detection plate in **Fig. S4A** were run on 0.8% agarose gel with the expected amplicon size of ~1.16-kb.

bioRxiv preprint doi: https://doi.org/10.1101/2024.12.06.627170; this version posted February 25, 2025. The copyright holder for this preprint (which was not certified by peer review) is the author/funder. This article is a US Government work. It is not subject to copyright under 17 USC 105 and is also made available for use under a CC0 license.

Table S1. Primers for AST cDNA synthesis and PCR amplification.

Primer Name	Primer Sequence (5′→3′)	Primer Use
Tag Fwd[A]	**CTGATCTAGAGGTACCGGATCC**	Tag acting as Fwd primer
AST cDNA	**CTGATCTAGAGGTACCGGATCC**AACATGTGGCAGGAAGTAGG	*env* AST cDNA primer
Env cDNA	**CTGATCTAGAGGTACCGGATCC**ACATCTAATTTGTCCACTGA	*env* cDNA primer
1849Tag+	**CTGATCTAGAGGTACCGGATCC**GATGACAGCATGTCAGGGAG	*gag/pol* AST cDNA primer
3996Tag+	**CTGATCTAGAGGTACCGGATCC**CATCTAGCTTTGCAGGATTCG	*pol/vif* AST cDNA primer
G00-Tag+	**CTGATCTAGAGGTACCGGATCC**GACTAGCGGAGGCTAGAAG	*gag* AST cDNA primer
AST-OF	**CTGATCTAGAGGTACCGGATCC**	*env* AST PCR1[B]
AST-OR	TGGTACTAGCTTGTAGCACCATCC	
AST-IF	AGCAGAACAATTTGCTGAGGGC	*env* AST PCR 2[C]
AST-IR	GTCATTGGTCTTAAAGGTACCTGAGG	
1849+	GGATCCGATGACAGCATGTCAGGGAG	*gag/pol* AST PCR1[D]
3500-	CTATTAAGTCTTTTGATGGGTCATAA	
1870+	GAGTTTTGGCTGAAGCAATGAG	*gag/pol* AST PCR2[E]
3410-	CTGTTAGTGGTATTACTTCTGTTAGTGCTT	
5270-	CTGACCCAAATGCCAGTCTC	*pol/vif* AST PCR1[F]
4133+	GGAAAAGGTCTATCTGGCATG	*pol/vif* AST PCR2[G]
5248-	TCTCCTGTATGCAGACCCCA	
G01	AGGGGTCGTTGCCAAAGA	*gag* AST PCR1[H]
G10	CAGTATTAAGCGGGGGAGAATT	*gag* AST PCR2[I]
G15	CTTTGCCACAATTGAAACACTT	
envB5out	TAGAGCCCTGGAAGCATCCAGGAAGT	*env* PCR1[J]
9538r	AGAGAGACCCAGTACAGGCAAAA	
envB5in	TTAGGCATCTCCTATGGCAGGAAGAAG	*env* PCR2[K]
9418r	CAAGCTCGATGTCAGCAGTTCT	

[A] Exogenous oligo-tag in black bold
[B] *env* AST PCR1: 94°C for 2 min, 45 cycles of 94°C for 15 sec, 60°C for 15 sec, and 68°C for 30 sec
[C] *env* AST PCR2: 94°C for 2 min, 40 cycles of 94°C for 15 sec, 60°C for 15 sec, and 68°C for 20 sec
[D] *gag/pol* AST PCR1: 94°C for 2 min, 45 cycles of 94°C for 15 sec, 60°C for 15 sec, and 68°C for 30 sec
[E] *gag/pol* AST PCR2: 94°C for 2 min, 40 cycles of 94°C for 15 sec, 60°C for 15 sec, and 68°C for 30 sec
[F] *pol/vif* AST PCR1: 94°C for 2 min, 45 cycles of 94°C for 15 sec, 60°C for 15 sec, and 68°C for 20 sec
[G] *pol/vif* AST PCR2: 94°C for 2 min, 40 cycles of 94°C for 15 sec, 60°C for 15 sec, and 68°C for 20 sec
[H] *gag* AST PCR1: 94°C for 2 min, 45 cycles of 94°C for 15 sec, 60°C for 15 sec, and 68°C for 25 sec
[I] *gag* AST PCR2: 94°C for 2 min, 40 cycles of 94°C for 15 sec, 60°C for 15 sec, and 68°C for 20 sec
[J] *env* PCR1: 94°C for 2 min, 45 cycles of 94°C for 15 sec, 60°C for 30 sec, and 68°C for 3 min
[K] *env* PCR2: 94°C for 2 min, 40 cycles of 94°C for 15 sec, 60°C for 30 sec, and 68°C for 20 sec

bioRxiv preprint doi: https://doi.org/10.1101/2024.12.06.627170; this version posted February 25, 2025. The copyright holder for this preprint (which was not certified by peer review) is the author/funder. This article is a US Government work. It is not subject to copyright under 17 USC 105 and is also made available for use under a CC0 license.

Table S2. Primers used for AST controls.

Primer Name	Primer Sequence (5′→3′)
AST-NEF2R	GTCATTGGTCTTAAAGGTACCTGAGG
AST-1586R	AGCAGAACAATTTGCTGAGGGC
AST-SEQ2R	GGTGAATATCCCTGCCTAACTCTAT
AST-SEQ2F	GGTTTAACATAACAAATTGGCTGTGGTATATAA
AST-SEQ3F	ATGGGTGGCAAGTGGTCAAA
Cloning Analysis Fwd	ACCTGCCAACCAAAGCGAGAAC
Cloning Analysis Rev	TCAGGGTTATTGTCTCATGAGCG
Rev16	ATGGGAGGGGCATACATTGCT
Rev17	CCTGGAGCTGTTTAATGCCCCAGAC
M13 Fwd	GTAAAACGACGGCCAGT
M13 Rev	CAGGAAACAGCTATGAC

bioRxiv preprint doi: https://doi.org/10.1101/2024.12.06.627170; this version posted February 25, 2025. The copyright holder for this preprint (which was not certified by peer review) is the author/funder. This article is a US Government work. It is not subject to copyright under 17 USC 105 and is also made available for use under a CC0 license.

Table S3. Digital PCR Primers and Probes.

Primer Name	Primer/Probe Sequence (5′→3′)	Primer Use
AST-Tag	**ATCTAGAGGTACCGGATCCAAC**	*env* AST
AST Rev-qPCR	TGATGAACATCTAATTTGTCCACTGA	
AST Probe	/56-FAM/AGCAATGT/ZEN/ATGCCCCTCCCA/3IaBkFQ/	
AST-Tag	**ATCTAGAGGTACCGGATCCACA**	*env*
env Fwd-qPCR	ACAAATTATAAACATGTGGCAGAAAGTAGG	
env Probe	/56-FAM/TGGGAGGGG/ZEN/CATACATTGCT/3IaBkFQ/	
LTR U5 Fwd	CTTAAGCCTCAATAAAGCTTGCC	LTR U5
LTR U5 Rev	GGATCTCTAGTTACCAGAGTC	
LTR U5 Probe	/5′HEX/AGTAGTGTG/ZEN/TGCCCGTCTG/3IaBkFQ/	
RRE Fwd	GCAGAGAGAAAAAAGAGC	RRE
RRE Rev	GCCTGTACCGTCAGC	
RRE Probe	/5′HEX/TTCCTTGGG/ZEN/TTCTTGGGAGCAG/3IaBkFQ/	
iCAD Fwd	TTTGGAAAGGACCAGCAAA	IN DNA Assay (iCAD)
iCAD Rev	CCTGCCATCTGTTTTCCA	
iCAD Probe	/56-FAM/AAAGGTGAA/ZEN/GGGGCAGTAGTAATACA/3IaBkFQ/	

Exogenous oligo-tag sequence in bold

bioRxiv preprint doi: https://doi.org/10.1101/2024.12.06.627170; this version posted February 25, 2025. The copyright holder for this preprint (which was not certified by peer review) is the author/funder. This article is a US Government work. It is not subject to copyright under 17 USC 105 and is also made available for use under a CC0 license.

Table S4. Sequencing Primers.

Primer Name	Primer Sequence (5′→3′)	Primer Use
AST-Seq2 Rev	GGTGAATATCCCTGCCTAACTCTAT	*env* AST
AST-Seq2 Fwd	GGTTTAACATAACAAATTGGCTGTGGTATATAA	
AST-Seq3 Fwd	ATGGGTGGCAAGTGGTCAAA	
AST-IF	AGCAGAACAATTTGCTGAGGGC	
AST-IR	GTCATTGGTCTTAAAGGTACCTGAGG	
2030+	TGTTGGAAATGTGGAAAGGAAGGAC	*gag/pol* AST
2600+	ATGGCCCAAAAGTTAAACAATGGC	
2610-	TTCTTCTGTCAATGGCCATTGTTTAAC	
3330-	TTGCCCAATTCAATTTTCCCACTAA	
Poli9D	AAAATTAGCAGGAMGATGGCCAG	*pol/vif* AST
Poli10B	TATTCATAGATTCYACTACTCCTTG	
4133+	GGAAAAGGTCTATCTGGCATG	
5248-	TCTCCTGTATGCAGACCCCA	
G30	CAGTAGCAACCCTCTATTGTGT	*gag* AST
G25	ATTGCTTCAGCCAAAACTCTTGC	
G10	CAGTATTAAGCGGGGGAGAATT	
G15	CTTTGCCACAATTGAAACACTT	
For16	TTTAATTGTGGAGGAGAATTTTTCTA	*env*
For17	AGCAGCAGGAAGCAGTATGGGCGC	
For18	CATATCAAATTGGCTGTGGTATAT	
Rev15	CTGCCATTTAACAGCAGTTGAGTTGA	
Rev16	ATGGGAGGGGCATACATTGCT	
envB5in	TTAGGCATCTCCTATGGCAGGAAGAAG	
envB5out	TAGAGCCCTGGAAGCATCCAGGAAGT	
9418r	CAAGCTCGATGTCAGCAGTTCT	

bioRxiv preprint doi: https://doi.org/10.1101/2024.12.06.627170; this version posted February 25, 2025. The copyright holder for this preprint (which was not certified by peer review) is the author/funder. This article is a US Government work. It is not subject to copyright under 17 USC 105 and is also made available for use under a CC0 license.

Table S5. Donor specific primers.

Primer Name	Primer Sequence (5′→3′)
2669_ASTI-F	AGCAGAACAATTTGCTGAG**R**GC
2669_ASTI-R	GTCATTGGTCTTAAAGGTACCTG**R**GG
2669 AST-R-qPCR	TGATGAACATCTAATT**A**GTCC**TT**TGA
2669_*env*-Tagged	**CTGATCTAGAGGTACCGGATCC**TGATGAACATCTAATT**A**GTCC**TT**TGA
1079_ASTR-qPCR	TGATGAACATCTAATTT**S**TCC**M**CTGA
1079_ *env* -Tagged	**CTGATCTAGAGGTACCGGATCC**ACATCTAATTT**S**TCC**M**CTGA
1683_AST-Tagged	**CTGATCTAGAGGTACCGGATCC**AACATGTGGCA**AG**AA**A**TAGG
1683_AST-OR	TGGTACTAGCTTGTAGCACCA**C**CC
1683_AST-IF	AGCAGAACAATTTGCTGAG**A**GC
1683_AST-R-qPCR	TGATGAACATCTAAT**CA**GTCC**G**CTGA
1683_ *env* -Tagged	**CTGATCTAGAGGTACCGGATCC**ACATCTAAT**CA**GTCC**G**CTGA
1683_ *env* F-qPCR	ACAAATTATAAACATGTGGCA**AG**AA**A**TAGG
291_AST-R	TGATGAACA**RY**TAATTT**K**TCC**TY**TGA
1508_AST-Tagged	**CTGATCTAGAGGTACCGGATCC**AA**T**ATGTGGCAG**GG**AGTAGG
1508_AST-R	TGATGAACA**K**CTAATTTGTCC**KYK**GA
1508_AST-P	/56-FAM/AGC**C**ATGTA/ZEN/TGCCCCTCCCA/3IaBkFQ/
3611_AST-Tagged	**CTGATCTAGAGGTACCGGATCC**AACATGTGGCA**AG**AAGTAGG
3611_AST-R	TGATG**C**ACA**G**CTAATT**Y**GTCC**T**CTGA
3611_AST-P	/56-FAM/A**G**GCAATGT/ZEN/ATGCCCCTCCCA/3IaBkFQ/
1226_AST-Tagged	**CTGATCTAGAGGTACCGGATCC**AACATGTGGCA**AC**AAGTAGG
1226_AST-R	TGATGAACA**G**CTAAT**C**TGTCC**T**C**G**GA
1775_AST-Tagged	**CTGATCTAGAGGTACCGGATCC**AACATGTGGCA**R**AAAGTAGG
1775_AST-R	TGATGAACAT**T**TAATTTGTCC**T**CTGA
477_AST-R	TGATGAACA**Y**CTAATTAGTCCACTGA

Exogenous oligo-tag sequence in black bold
Bold red nucleotide reflects donor specific base
Degenerate bases: R (A/G), S (S/G), M (A/C), Y (C/T), and K (G/T) following IUPAC nucleotide code

bioRxiv preprint doi: https://doi.org/10.1101/2024.12.06.627170; this version posted February 25, 2025. The copyright holder for this preprint (which was not certified by peer review) is the author/funder. This article is a US Government work. It is not subject to copyright under 17 USC 105 and is also made available for use under a CC0 license.

Table S6. Levels of HIV-1 AST in ART-treated and untreated donors.

Participant Identifier (PID)	Duration on ART at sampling	Estimated number of infected PBMC assayed[B]	Number of HIV-1 AST molecules detected	Number of HIV-1 AST molecules per 100 infected PBMC
1079	12.8 years	80	9	11
1683	5.4 years	188	32	17
2669	4.3 years	90	19	21
	5.5 years	65	20	31
	2 weeks[A]	88	34	39
	1 month[A]	45	31	69
	**Median**	**84**	**26**	**26**
	**IQR**	**60-115**	**17-33**	**16-47**
291	0	104	2	2
477	0	83	2	2
1508	0	22	1	5
1775	0	40	13	33
3611	0	244	1	≤0.4[C]
	**Median**	**83**	**2**	**2**
	**IQR**	**31-174**	**1-8**	**1-19**

[A] After 5.5 years on ART, the participant had an unexpected ART interruption for approximately 4 weeks They reinitiated ART with the first timepoint post-ART interruption at 2 weeks with low but detectable HIV-1 plasma viremia. Then 1 month post-ART interruption with plasma viremia suppressed

[B] Estimated the number of HIV DNA levels using the integrase cell-associated single-copy DNA (iCAD) assay (*24*)

[C] Detectable but below the cut-off
